# Limitations of Binary Classification for Long-Horizon Diagnosis Prediction and Advantages of a Discrete-Time Time-to-Event Approach: Empirical Analysis

**DOI:** 10.2196/62985

**Published:** 2025-03-27

**Authors:** De Rong Loh, Elliot D Hill, Nan Liu, Geraldine Dawson, Matthew M Engelhard

**Affiliations:** 1 Duke-NUS Medical School, 8 College Road, Singapore, 169857, Singapore; 2 Department of Biostatistics and Bioinformatics, Duke University School of Medicine, Durham, NC, United States; 3 Department of Psychiatry and Behavioral Sciences, Duke University School of Medicine, Durham, NC, United States

**Keywords:** machine learning, artificial intelligence, deep learning, predictive models, practical models, early detection, electronic health records, right-censoring, survival analysis, distributional shifts

## Abstract

**Background:**

A major challenge in using electronic health records (EHR) is the inconsistency of patient follow-up, resulting in right-censored outcomes. This becomes particularly problematic in long-horizon event predictions, such as autism and attention-deficit/hyperactivity disorder (ADHD) diagnoses, where a significant number of patients are lost to follow-up before the outcome can be observed. Consequently, fully supervised methods such as binary classification (BC), which are trained to predict observed diagnoses, are substantially affected by the probability of sufficient follow-up, leading to biased results.

**Objective:**

This empirical analysis aims to characterize BC’s inherent limitations for long-horizon diagnosis prediction from EHR; and quantify the benefits of a specific time-to-event (TTE) approach, the discrete-time neural network (DTNN).

**Methods:**

Records within the Duke University Health System EHR were analyzed, extracting features such as *ICD-10* (*International Classification of Diseases, Tenth Revision*) diagnosis codes, medications, laboratories, and procedures. We compared a DTNN to 3 BC approaches and a deep Cox proportional hazards model across 4 clinical conditions to examine distributional patterns across various subgroups. Time-varying area under the receiving operating characteristic curve (AUC_t_) and time-varying average precision (AP_t_) were our primary evaluation metrics.

**Results:**

TTE models consistently had comparable or higher AUC_t_ and AP_t_ than BC for all conditions. At clinically relevant operating time points, the area under the receiving operating characteristic curve (AUC) values for DTNN_YOB≤2020_ (year-of-birth) and DCPH_YOB≤2020_ (deep Cox proportional hazard) were 0.70 (95% CI 0.66‐0.77) and 0.72 (95% CI 0.66‐0.78) at *t*=5 for autism, 0.72 (95% CI 0.65‐0.76) and 0.68 (95% CI 0.62‐0.74) at *t*=7 for ADHD, 0.72 (95% CI 0.70‐0.75) and 0.71 (95% CI 0.69‐0.74) at *t*=1 for recurrent otitis media, and 0.74 (95% CI 0.68‐0.82) and 0.71 (95% CI 0.63‐0.77) at *t*=1 for food allergy, compared to 0.6 (95% CI 0.55‐0.66), 0.47 (95% CI 0.40‐0.54), 0.73 (95% CI 0.70‐0.75), and 0.77 (95% CI 0.71‐0.82) for BC_YOB≤2020_, respectively. The probabilities predicted by BC models were positively correlated with censoring times, particularly for autism and ADHD prediction. Filtering strategies based on YOB or length of follow-up only partially corrected these biases. In subgroup analyses, only DTNN predicted diagnosis probabilities that accurately reflect actual clinical prevalence and temporal trends.

**Conclusions:**

BC models substantially underpredicted diagnosis likelihood and inappropriately assigned lower probability scores to individuals with earlier censoring. Common filtering strategies did not adequately address this limitation. TTE approaches, particularly DTNN, effectively mitigated bias from the censoring distribution, resulting in superior discrimination and calibration performance and more accurate prediction of clinical prevalence. Machine learning practitioners should recognize the limitations of BC for long-horizon diagnosis prediction and adopt TTE approaches. The DTNN in particular is well-suited to mitigate the effects of right-censoring and maximize prediction performance in this setting.

## Introduction

Electronic health records (EHR) are a rich source of data that can be used to develop effective clinical prediction models to improve patient care [1]. However, a major challenge is that patients have inconsistent follow-ups, leading to right-censored outcomes, and follow-up length typically depends on observed covariates. This challenge is exacerbated in long-horizon event prediction, such as prediction of an autism and attention-deficit/hyperactivity disorder (ADHD) diagnosis early in life, because many patients are lost to follow-up before the outcome can be observed. Consequently, the probability of observing a diagnosis depends not only on the probability of diagnosis but also on the probability of sufficient follow-up (ie, the probability that diagnosis occurs before censoring). As a result, binary classification (BC) models trained to predict observed diagnoses are substantially affected by the probability of sufficient follow-up unless filtering strategies are carefully applied [2].

A common filtering strategy to mitigate this effect is to exclude all individuals with insufficient follow-up. However, this is not feasible for many long-term prediction tasks. For example, sufficient follow-up for ADHD would extend into adolescence and adulthood; therefore, this criterion would preclude the development of early ADHD prediction models. Even in cases where such a criterion is feasible, it can significantly reduce the sample size available for learning and introduce systematic biases [3], as it tends to exclude subpopulations with shorter follow-up, including disadvantaged groups.

Time-to-event (TTE; ie, survival analysis) methods are the natural alternative, as they are designed for right-censored outcomes. Various versions of classification trees and random forests [4,5], Bayesian networks [6,7], Cox proportional hazards regression [8] and neural networks [9,10] have been applied to survival data with mixed success, and have been adapted to the EHR setting [11]. Deep learning [12] models such as DeepSurv [13] or deep Cox proportional hazards (DCPHs), which follow the Cox proportional hazards framework but uses a neural network to predict the log-hazard ratio, have become popular for EHR prediction tasks. Neural network-based TTE approaches are advantageous because they can efficiently process large, unstructured, high-dimensional inputs and capture complex nonlinear relationships between features and outcomes.

However, common TTE approaches also have limitations relevant to long-horizon diagnosis prediction. Unlike in survival analysis, the event of interest never occurs in most patients, and typically we are more concerned with predicting diagnosis probability than predicting diagnosis timing. Consequently, approaches that predict the probability of diagnosis separately from its timing [14] are well-suited for long-horizon diagnosis prediction, whereas DCPH and other approaches that assume relative likelihood does not change over time are less appropriate. These considerations motivate our current work to use a discrete-time neural network (DTNN), which combines the benefits of BC and TTE approaches.

First, the DTNN offers significant flexibility. Specifically, it does not assume a particular parametric form for the event time density, and in particular, allows the effect of covariates on risk to vary across the time horizon. Second, the DTNN predicts the probability of no-event within the time horizon, which is useful in diagnosis prediction where the event of interest may often not occur. For these reasons, we have found DTNN to be advantageous in our work.

In this paper, we examine the advantages of the DTNN approach compared to BC and DCPH across 4 long-horizon, EHR-based event prediction tasks. We hypothesize that the DTNN approach will yield higher discrimination performance and more accurate likelihood predictions compared to BC even after common filtering strategies are applied due to the inability of BC to disentangle the probability of diagnosis from that of insufficient follow-up. We further hypothesize that DTNN performance will be higher than DCPH, and DTNN predictions will better reflect real-world clinical prevalence and patterns. The code for our work is available online [15].

## Methods

### Ethical Considerations

All study procedures were approved by the Duke Health Institutional Review Board (Pro00111224) and comply with institutional policies and federal regulations. A waiver of participant consent was approved due to the minimal risk posed by study procedures and the infeasibility of obtaining consent in a large retrospective cohort. No compensation was provided to the participants. Identifiers were omitted during analysis, which was executed within the Duke PACE (Protected Analytics Computing Environment), a highly secure virtual network space designed for protected health information.

### Cohort Identification

Analyses were based on inpatient and outpatient encounters within the Duke University Health System (DUHS), a large academic medical center based in Durham, NC. DUHS provides care to approximately 85% of children in Durham and surrounding Durham County, which has a diverse population with varying demographic and socioeconomic status [16]. Records were extracted from the current (2014‐2023) DUHS EHR, which is based on the platform developed by Epic.

Study inclusion criteria were the following: (1) date of birth between January 1, 2014 and October 29, 2022; and (2) ≥1 visit within the DUHS before aging 30 days. DUHS encounters between January 1, 2014 and June 2, 2023 were extracted for individuals meeting these criteria. See Figure S1 in Multimedia Appendix 1 for the distribution of year of birth for this identified cohort.

### Diagnosis Identification

We focused on 4 clinical diagnoses: autism spectrum disorder (autism), ADHD, recurrent otitis media (ROM), and food allergy (FA). We used computable phenotypes previously established within DUHS [17] or formulated in consultation with clinicians. The classification criteria are provided in Tables S1 and S2 in Multimedia Appendix 1.

### Experimental Setup

**Figure 1.**
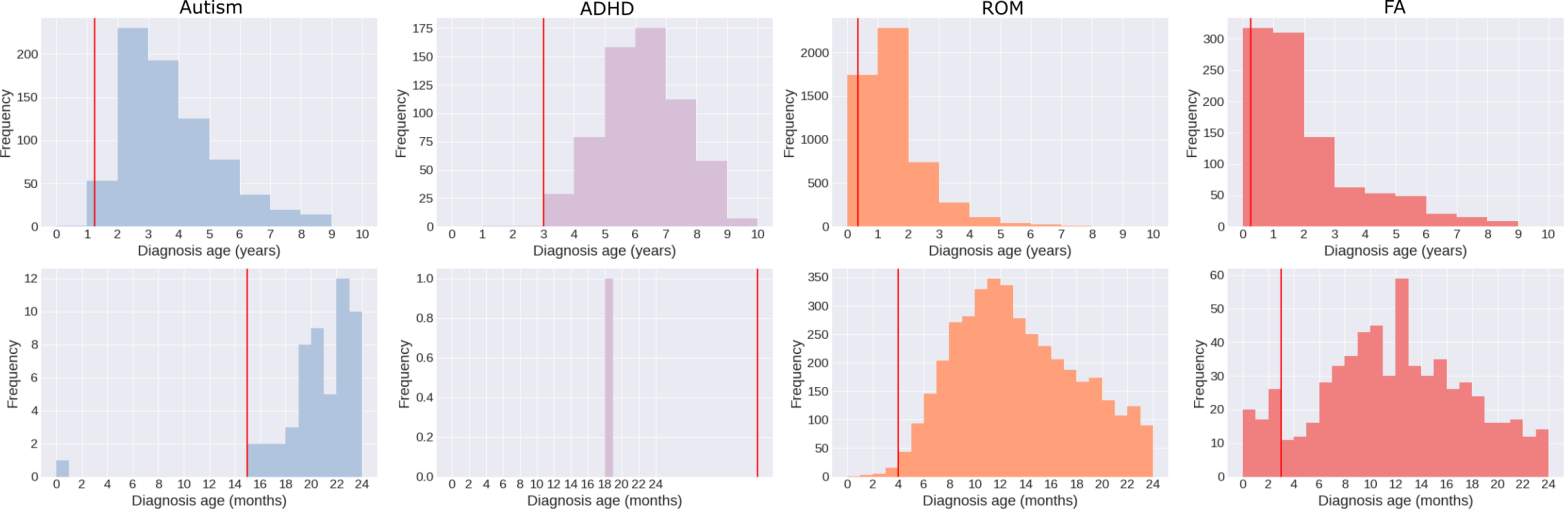
Distribution of observed diagnosis ages in years (upper panel) and months (lower panel). Children with diagnoses before respective diagnosis age cutoffs (marked by the red line) were excluded. Note that there were 2 ADHD diagnoses before the age cutoff of 3 years. ADHD: attention-deficit/hyperactivity disorder; FA: food allergy; ROM: recurrent otitis media.

BC models predicting observed diagnoses are significantly influenced by adequate follow-up probabilities, requiring meticulous filtering strategies. We first conducted baseline experiments to establish the performance of BC models with and without exclusion criteria based on year-of-birth (YOB) or follow-up length. Correspondingly, we have 3 models trained on different cohort subsets, which are denoted as BC_YOB≤2020_, BC_YOB≤2018_, and BC_t≥5_ (where t denotes follow-up length). The upper limit of the dataset for the prediction tasks was capped at 2020 due to the rarity of autism and ADHD diagnoses before the age of 2 years (Figure 1). For subset YOB ≤2018, we excluded all children who were age younger than 5 years at the end of our observation window to limit effects of early censoring on model predictions. For subset *t*≥5, we excluded all children with <5 years of follow-up as a more aggressive measure; note that this subset overlaps the subset YOB≤2018. Next, we introduced 2 TTE models, namely DTNN_YOB≤2020_ and DCPH_YOB≤2020_, and evaluated their performance against the 3 BC approaches. To summarize, we explored the effect of each setup when training the corresponding model to predict each of the 4 conditions, yielding 20 models in total.

Our features were based on encounters taking place before the following predefined, condition-specific prediction ages: 15 months, 3 years, 4 months, and 3 months for autism, ADHD, ROM, and FA, respectively (Figure 1). These ages were chosen to be clinically useful prediction times that were earlier than most observed diagnoses. Individuals diagnosed or censored before these cutoffs were excluded from the analysis. To prevent temporal data leakage, the events used for prediction were limited to those taking place before the first diagnosis code (*ICD-10* [*International Classification of Diseases, Tenth Revision*]) associated with the outcome of interest. The distribution of censoring ages can be found in Figure S2 in Multimedia Appendix 1.

The use of predefined diagnosis age cutoffs was a deliberate design decision. First, we aimed to demonstrate the predictive value of detection models based solely on EHR data collected from early ages [17]. Second, using fixed age-offs standardizes the data collection period for all individuals, which simplifies analysis and ensures consistency across the dataset. This approach allows us to focus on understanding model performance across various clinical conditions without the additional complexity of time-dependent updates.

For each diagnosis, the dataset was partitioned randomly, allocating 60% for training, 20% for validation, and 20% for testing.

### Model Development

#### Overview

Each observation was represented by the triplet {X,T,S}, where $X\subseteq R^{d}$ is a *d*-dimensional feature vector, $T\in (0,E_{max}]$ is an observed event or censoring time over a finite time horizon, and S∈{0,1} indicates whether *T* is a right-censoring time (*S=0*) or an event time (*S=1*). The observed time *T* is the minimum of the event time *E* and the right-censoring time *C*, that is, T=min(E,C).

**Figure 2.**
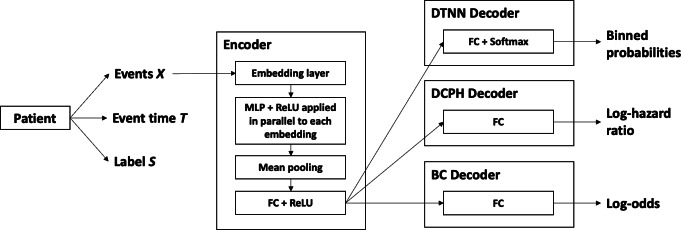
Model architectures of DTNN, DCPH, and BC. BC: binary classification; DCPH: deep Cox proportional hazard; DTNN: discrete-time neural network; FC: fully connected; MLP: multilayer perceptron; ReLU: rectified linear unit.

The model selection process began with experimenting with different combinations of fully connected layers and transformer architectures. See Figure 2 for the final model architectures.

#### Pretraining Medical Concept Embeddings

Patient histories were represented as timestamped sequences of DUHS EHR events, including *ICD-10* diagnosis codes, medications (RxNorm [18] codes), procedures (Current Procedural Terminology [19] codes), and laboratories (Logical Observation Identifiers Names and Codes [20] codes). Events were mapped to corresponding Word2Vec embeddings, which were learned by training the model on these event sequences to capture contextual relationships between codes. The model used a Continuous Bag of Words approach with negative sampling, producing embeddings of size 256. Padding and out-of-vocabulary indices were also included and mapped to a vector of zeroes. Table S3 in Multimedia Appendix 1 details the hyperparameters used during the training process.

#### Encoder Architecture

The BC and TTE models all shared a common underlying encoder architecture comprised of (1) an embedding layer, (2) a fully connected layer with rectified linear unit activation applied in parallel to each embedding, (3) a global mean pooling layer, and (4) a fully connected layer with rectified linear unit activation. The embedding layer was initialized with frozen pretrained weights from the Word2Vec model. The sequence length was fixed at 512. Shorter sequences were padded, while longer sequences were truncated by selecting the most recent events preceding the age cutoff for a given model. The mean pooling layer was applied across the sequence dimension, resulting in a single fixed-length vector with dimension equal to that of the embeddings.

#### Prediction Head

In DTNN, the prediction head was a single fully connected hidden layer with Softmax activation, producing a probability distribution across multiple bins. The bin boundaries can be found in Table S4 in Multimedia Appendix 1. Under the common assumption of noninformative right-censoring, we may ignore the censoring density and optimize the likelihood P(t, s |x;θ) over the observed data $D={\left\{x_{i},t_{i},s_{i}\right\}}_{i=1}^{N}$ by minimizing the following loss:


$$L_{MLE}\left(\theta \right)=-\left(s_{i}\log p_{\theta }\left(t_{i}|x_{i}\right)+\left(1-s_{i}\right)\log P_{\theta }\left(t_{i}|x_{i}\right)\right)$$

where $P_{\theta }$ is the survival function associated with $p_{\theta }$ and *T* has been discretized such that each $t_{i}$ indicates which interval contains min(E,C).

In BC and DCPH, the prediction head was a fully connected hidden layer predicting the log-odds and log-hazard ratio, respectively, with corresponding binary cross entropy or cox negative partial log-likelihood [21] loss. Whereas BC directly predicts the probability that diagnosis will be observed (by applying the logistic function to the predicted log-odds), with DCPH this probability may be derived from the predicted log-hazard ratio and baseline hazard function. Note that for BC, we assumed a constant predicted probability irrespective of the time point.

#### Hyperparameter Tuning

The hyperparameters, consisting of learning rate and weight decay, were then chosen through a grid search to minimize loss on the validation set (Table S5 in Multimedia Appendix 1). These optimized models were subsequently used for evaluation on the test set.

### Model Evaluation

#### Calibration Curves

The BC models were evaluated using the probability calibration module from the *scikit-learn* library [22], while the TTE models were evaluated by comparing the observed probabilities (ie, estimated survival probabilities of the Kaplan-Meier estimator) and the predicted probabilities at selected time intervals [23].

#### Performance Metrics

Our primary evaluation metrics were the time-varying area under the receiving operating characteristic curve (AUC_t_) and time-varying average precision (AP_t_) [24], which quantify the model’s ability to discriminate between individuals diagnosed before the age t (positives; S*=1,* t≤t) and individuals remaining event-free beyond age t (negatives; t>t). This time-dependent approach is necessary due to censoring, which prevents many diagnoses from being observed. In contrast, the standard area under the receiving operating characteristic curve (AUC) and average precision (AP) do not differentiate between nondiagnosed individuals with short versus long follow-up, making them unsuitable for evaluating predicted diagnosis probabilities.

Harrell concordance index [25] was also used to quantify the agreement between likelihood predictions and event times. This metric quantifies the model’s ability to discriminate between individuals diagnosed earlier and those diagnosed later or not at all.

For each metric, we computed the 95% CI of the distribution over performance obtained from 100 bootstrap samples in the test set.

As we were unable to directly assess the accuracy of the predicted probabilities because diagnoses were not fully observed in the dataset, we instead contextualized them and reasoned about their correctness by analyzing the corresponding published trends.

### Subgroup Analysis

To explore possible differential effects of each model setup on specific demographics, we analyzed model predictions and performance in subgroups defined by YOB, follow-up length (ie, age at censoring), sex, race, and insurance. Biological sex was classified as male or female. Race was categorized into the following groups: Asian, Black or African American, White, unavailable, and other. Insurance status was separated into public, private, and other categories.

To assess the performance of our models on out-of-distribution (OOD) data, we extended the evaluation to include children born after 2018 and individuals with a follow-up duration of <5 years for the YOB and follow-up length plots, respectively. For the YOB plots, 2019 and 2020 were designated as OOD years for BC_YOB≤2018_. Since BC_t≥5_ also fulfilled the YOB≤2018 criteria, the same years were, by extension, considered OOD. Similarly, for the follow-up length plots, individuals with a follow-up duration of ≥5 years were categorized as in-distribution, while those with <5 years were classified as OOD.

### Semisynthetic ROM Dataset

To further explore the effect of early censoring on each method’s ability to predict diagnosis probability, we simulated early censoring for ROM cases. Unlike ADHD, most ROM diagnoses were observed rather than censored due to the earlier age of diagnosis. Leveraging prior knowledge of true ROM labels, we introduced artificial censoring by scaling the true censoring distribution such that the maximum age is at 1.2 years to mimic the ADHD scenario. Generating a semisynthetic ROM dataset served 2 purposes: reproducing earlier findings on BC limitations with censored data and demonstrating DTNN model performance under such conditions. Additional DTNN and BC models were trained on this semisynthetic train dataset and subsequently evaluated on the original test dataset.

This study follows the Consolidated Reporting of Machine Learning Studies guidelines (Checklist 1) [26].

## Results

### Patient Characteristics

Records for 57,701 unique patients meeting study criteria were initially extracted. After excluding children born after 2020, the evaluation dataset comprised 43,536 patients (Table 1). Based on the respective diagnosis age cutoffs (Figure 1), we further excluded 1 individual with autism as an outlier due to a diagnosis within the first month of birth, along with 2 individuals with ADHD, 25 individuals with ROM, and 70 with FA. Additionally, individuals with censoring ages preceding the age cutoffs were excluded: 9332 from the autism dataset, 17,691 from the ADHD dataset, 6171 from the ROM dataset, and 5847 from the FA dataset.

**Table 1.** Patient demographics.

Variable and category or value		All	Autism	ADHD^a^	ROM^b^	FA^c^
Total, n (%)		43,536 (100)	749 (1.7)	618 (1.4)	5201 (11.9)	916 (2.1)
Sex						
Male, n (%)		22,583 (51.9)	590 (78.8)	432 (69.9)	2951 (56.7)	544 (59.4)
Female, n (%)		20,953 (48.1)	159 (21.2)	186 (30.1)	2250 (43.3)	372 (40.6)
Chi-square (*df*)		N/A^d^	221.9 (1)	79.7 (1)	58.8 (1)	21.5 (1)
*P* value		N/A	<.001	<.001	<.001	<.001
Race, n (%)						
Asian		1835 (4.2)	23 (3.1)	8 (1.3)	145 (2.8)	63 (6.9)
Black or African American		13,132 (30.2)	272 (36.3)	206 (33.3)	1226 (23.6)	278 (30.3)
White		18,681 (42.9)	266 (35.5)	326 (52.8)	2936 (56.5)	418 (45.6)
Unavailable		3874 (8.9)	57 (7.6)	29 (4.7)	390 (7.5)	45 (4.9)
Other		6014 (13.8)	131 (17.5)	49 (7.9)	504 (9.7)	112 (12.2)
Chi-square (*df*)		N/A	22.1 (4)	55 (4)	521.9 (4)	44.7 (4)
*P* value		N/A	<.001	<.001	<.001	<.001
Insurance, n (%)						
Public		23,262 (53.4)	431 (57.5)	326 (52.8)	2011 (38.7)	319 (34.8)
Private		20,127 (46.2)	316 (42.2)	288 (46.6)	3178 (61.1)	596 (65.1)
Other		147 (0.3)	2 (0.3)	4 (0.6)	12 (0.2)	1 (0.1)
Chi-square (*df*)		N/A	4.3 (2)	0.7 (2)	571.1 (2)	141.7 (2)
*P* value		N/A	.12	.69	<.001	<.001

a ADHD: attention-deficit/hyperactivity disorder.

b ROM: recurrent otitis media.

c FA: food allergy.

d N/A: not applicable.

Male-to-female ratios were 3.7 for autism, 2.3 for ADHD, 1.3 for ROM, and 1.5 for FA. All diagnoses were associated with sex (*P*<.001) and racial status (*P*<.001). ROM and FA were associated with insurance status (*P*<.001), but autism and ADHD were not (*P*=.12 and *P*=.69, respectively). Private insurance rates were 3178/5201 (61.1%) and 596/916 (65.1%) in the ROM and FA groups, respectively, compared to 316/749 (42.2%) and 288/618 (46.6%) in the autism and ADHD groups, respectively.

The mean age at diagnosis for autism and ADHD was 3.75 years and 6.22 years, respectively, higher than that for ROM and FA, which were 1.57 years and 2.01 years, respectively (Figure 1).

### Analysis of Performance Metrics

**Figure 3.**
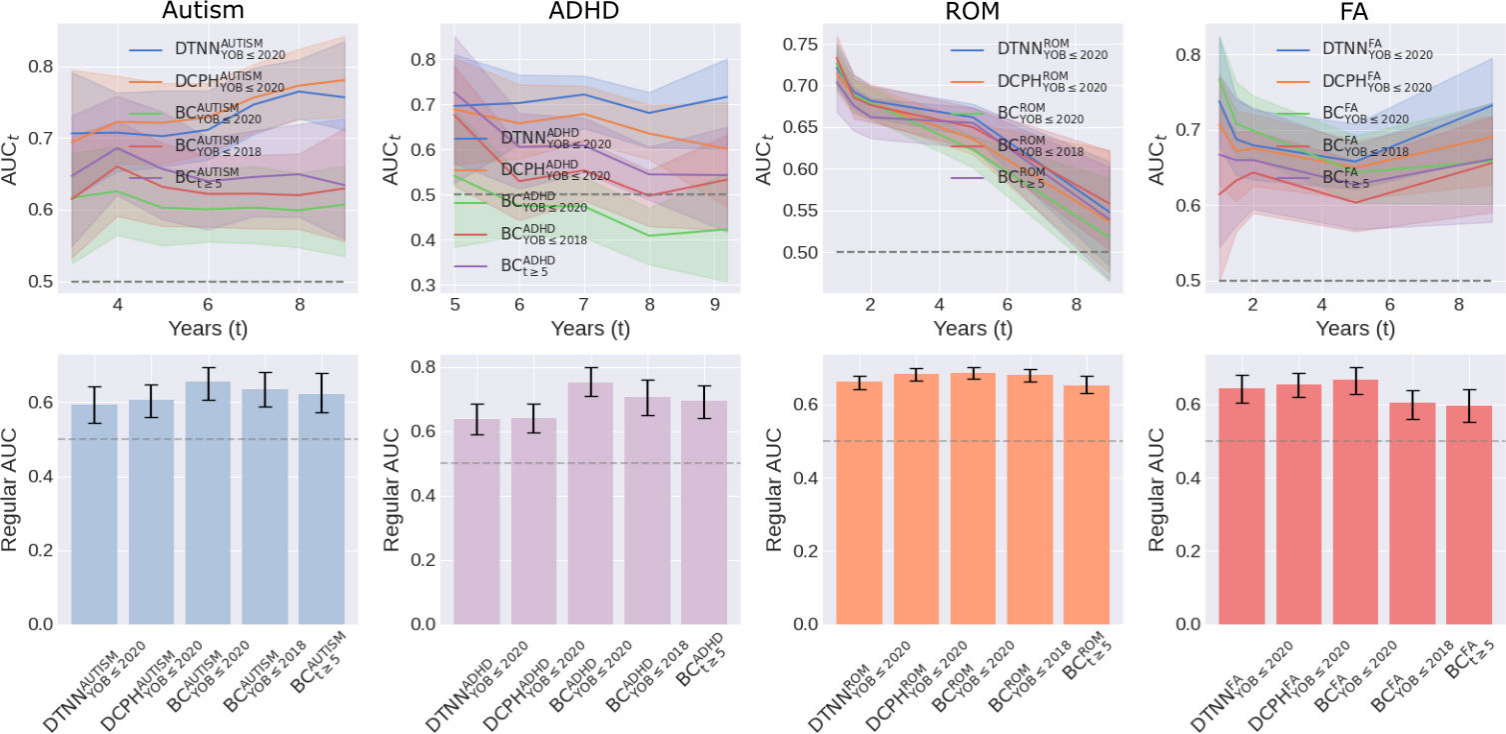
Comparison of AUC_t_ (solid lines) and regular AUC (bar graphs). ADHD: attention-deficit/hyperactivity disorder; AUC: area under the receiving operating characteristic curve; AUC_t_: time-varying area under the receiving operating characteristic curve; BC: binary classification; DCPH: deep Cox proportional hazard; DTNN: discrete-time neural network; FA: food allergy; ROM: recurrent otitis media; t: t denotes follow-up length; YOB: year-of-birth.

In general, the TTE models consistently matched or outperformed BC models with higher AUC_t_ values across all conditions (Figure 3 and Table S6 in Multimedia Appendix 1). At clinically relevant operating time points, the AUC values for DTNN_YOB≤2020_ and DCPH_YOB≤2020_ were 0.70 (95% CI 0.66‐0.77) and 0.72 (95% CI 0.66‐0.78) at t=5 for autism, 0.72 (95% CI 0.65‐0.76) and 0.68 (95% CI 0.62‐0.74) at t=7 for ADHD, 0.72 (95% CI 0.70‐0.75) and 0.71 (95% CI 0.69‐0.74) at t=1 for ROM, and 0.74 (95% CI 0.68‐0.82) and 0.71 (95% CI 0.63‐0.77) at t=1 for FA, compared to 0.60 (95% CI 0.55‐0.66), 0.47 (95% CI 0.40‐0.54), 0.73 (95% CI 0.70‐0.75), and 0.77 (95% CI 0.71‐0.82) for BC_YOB≤2020_, respectively.

Conversely, the regular AUC values for BC_YOB≤2020_ were consistently higher than those for DTNN_YOB≤2020_ and DCPH_YOB≤2020_. Notably, a statistically significant difference (*P*<.05) was observed in the ADHD prediction task (${\text{BC}}_{\text{YOB≤2020}}^{\text{ADHD}}$: AUC 0.75, 95% CI 0.71‐0.80; ${\text{DTNN}}_{\text{YOB≤2020}}^{\text{ADHD}}$: AUC 0.64, 95% CI 0.59‐0.69; ${\text{DCPH}}_{\text{YOB≤2020}}^{\text{ADHD}}$: AUC 0.64, 95% CI 0.60‐0.69). With filtering, BC_YOB≤2020_ and BC_t≥5_ exhibited decreased regular AUC, with the latter experiencing a larger decline.

The regular AP and AP_t_ exhibited similar trends as described above, with higher AP_t_ but lower regular AP for TTE models (Figure S3 and Table S7 in Multimedia Appendix 1). However, direct comparison and interpretation are difficult due to the variation in test prevalence across different datasets. The concordance index, comparing ordered predicted event probabilities with observed event times, further demonstrates that the TTE models consistently performed as well as or better than the BC models (Table S8 in Multimedia Appendix 1). In particular, DTNN_YOB≤2020_ and DCPH_YOB≤2020_ achieved 0.656 and 0.667 for autism, 0.682 and 0.657 for ADHD, as compared to 0.629 and 0.558 for BC_YOB≤2020_, respectively.

**Figure 4.**
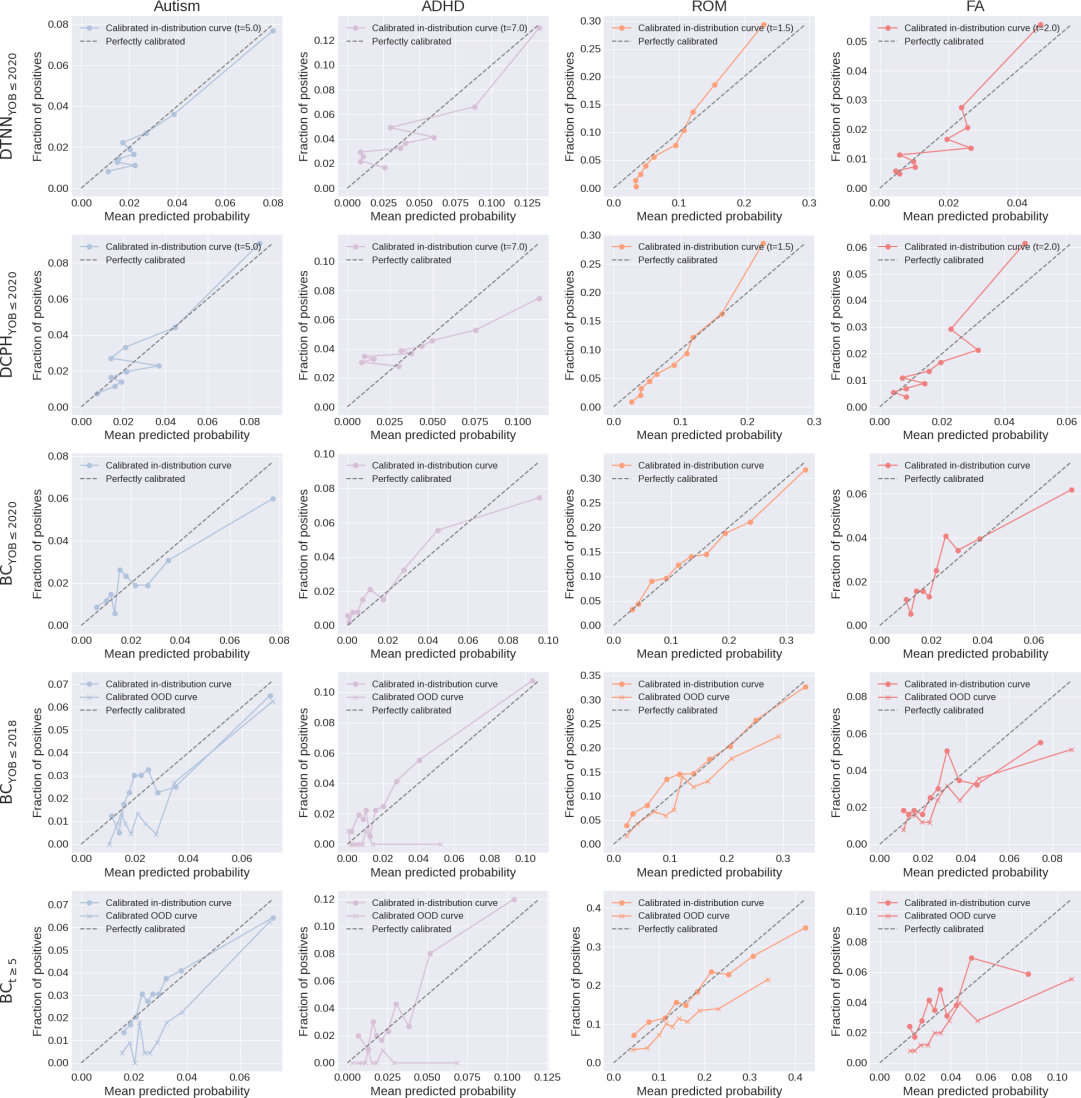
Calibration analysis. The predicted probabilities were compared with observed event rates across different probability bins, using Kaplan-Meier estimates for the TTE models and true binary outcomes for the BC models. OOD curves (ie, years 2019 and 2020) were also added for BC_YOB≤2018_ and BC_t≥5_. ADHD: attention-deficit/hyperactivity disorder; BC: binary classification; DCPH: deep Cox proportional hazard; DTNN: discrete-time neural network; FA: food allergy; OOD: out-of-distribution; ROM: recurrent otitis media; t: t denotes follow-up length; TTE: time-to-event.

The predicted probabilities for all models closely align with the observed estimates for in-distribution years, demonstrating overall good calibration, while OOD curves (ie, years 2019 and 2020) for BC_YOB≤2018_ and BC_t≥5_ show poor calibration (Figure 4).

### Semisynthetic Censoring Experiment Results

**Figure 5.**
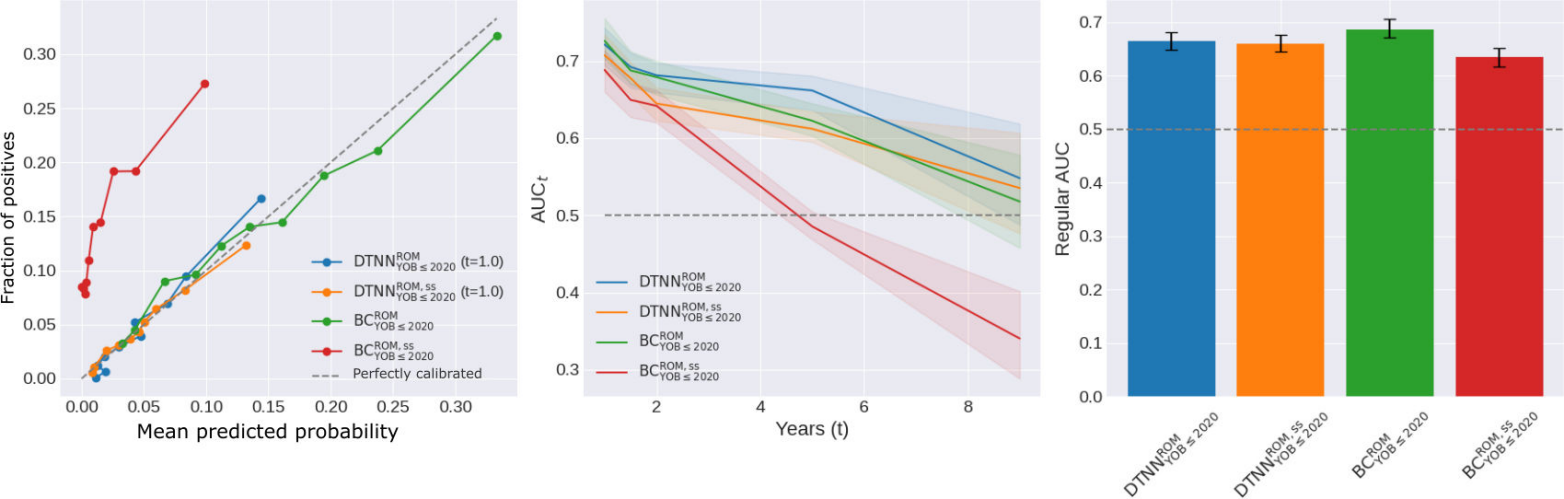
Comparison of performance metrics evaluated on the original test set between BC and DTNN models trained on original and semisynthetic ROM train datasets. AUC: area under the receiving operating characteristic curve; AUC_t_: time-varying area under the receiving operating characteristic curve; BC: binary classification; DTNN: discrete-time neural network; ROM: recurrent otitis media; YOB: year-of-birth.

The ${\text{DTNN}}_{\text{YOB≤2020}}^{\text{ROM, ss}}$ performance remained comparable to ${\text{DTNN}}_{\text{YOB≤2020}}^{\text{ROM}}$ and ${\text{BC}}_{\text{YOB≤2020}}^{\text{ROM}}$, exhibited good calibration, AUC_t_ and regular AUC values. However, ${\text{BC}}_{\text{YOB≤2020}}^{\text{ROM, ss}}$ displayed worse calibration due to underprediction, and had lower AUC_t_ and regular AUC values (Figure 5). Note that comparing performances beyond 1.2 years would be unfair, as those observed times were not available for model learning during training in the semisynthetic setup.

### Subgroup Analyses

**Figure 6.**
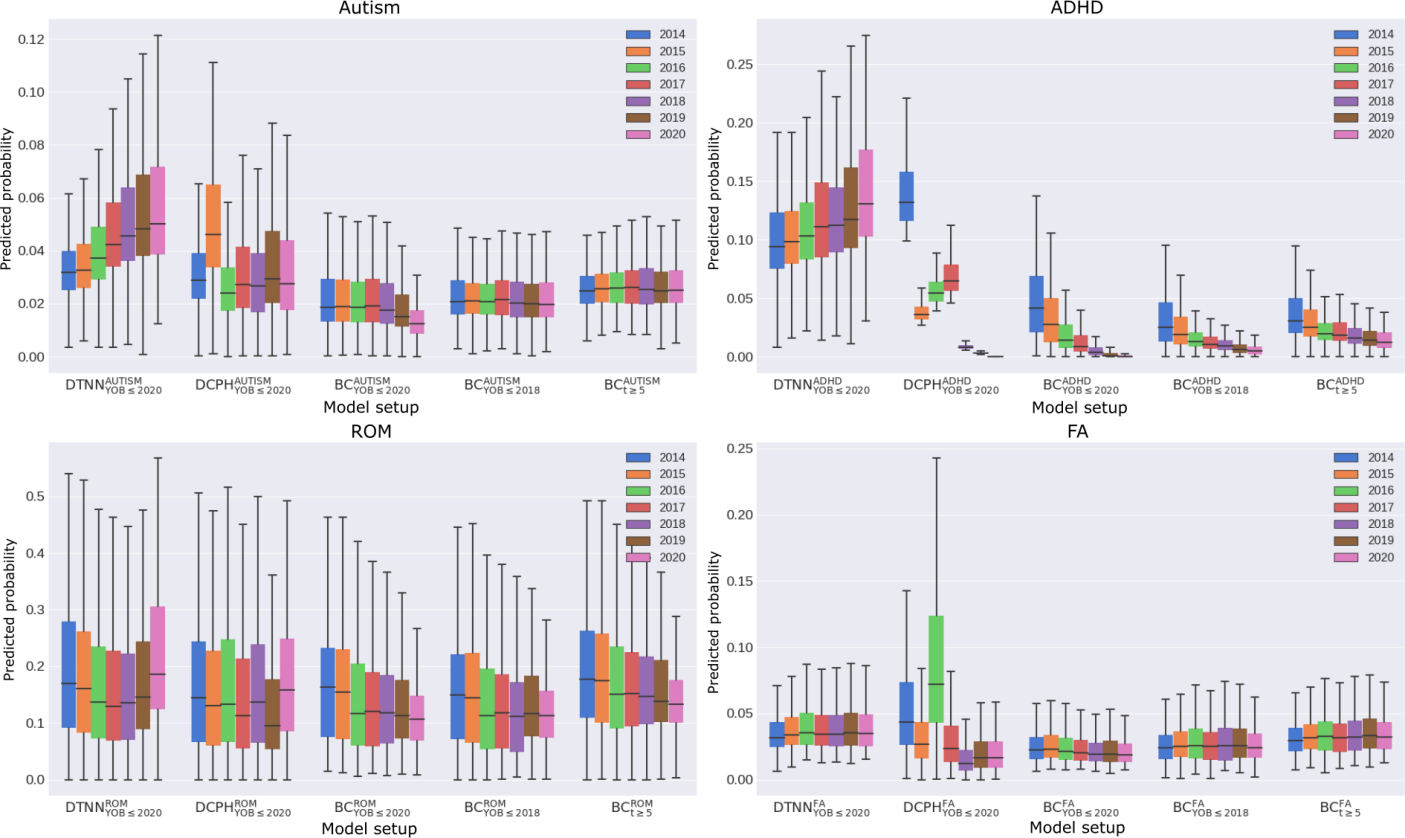
Grouped analysis of predicted probability distributions by year-of-birth. ADHD: attention-deficit/hyperactivity disorder; BC: binary classification; DCPH: deep Cox proportional hazard; DTNN: discrete-time neural network; FA: food allergy; ROM: recurrent otitis media; t: t denotes follow-up length; YOB: year-of-birth.

Probabilities predicted by BC_YOB≤2020_ decreased over time across all conditions. This trend was less pronounced for BC_YOB≤2018_ and BC_t≥5_ (Figure 6). In contrast, the probabilities predicted by DTNN_YOB≤2020_ for autism and ADHD showed a consistent yearly increase. For ROM, predicted probabilities declined from 2014 to 2017, then increased from 2018 onward. For FA, predicted probabilities modestly increased from 2014 to 2015, then stabilized at approximately 3.4%‐3.5% in subsequent years. The results for DCPH_YOB≤2020_ were heterogeneous.

We expanded our YOB subgroup analysis to include 2019 and 2020 to evaluate BC model behaviours during these OOD years (Figure 6). BC_t≥5_ exhibited a modest decrease in predicted probabilities across all the conditions, more pronounced in 2020 than in 2019, while BC_YOB≤2018_ remained relatively stable.

**Figure 7.**
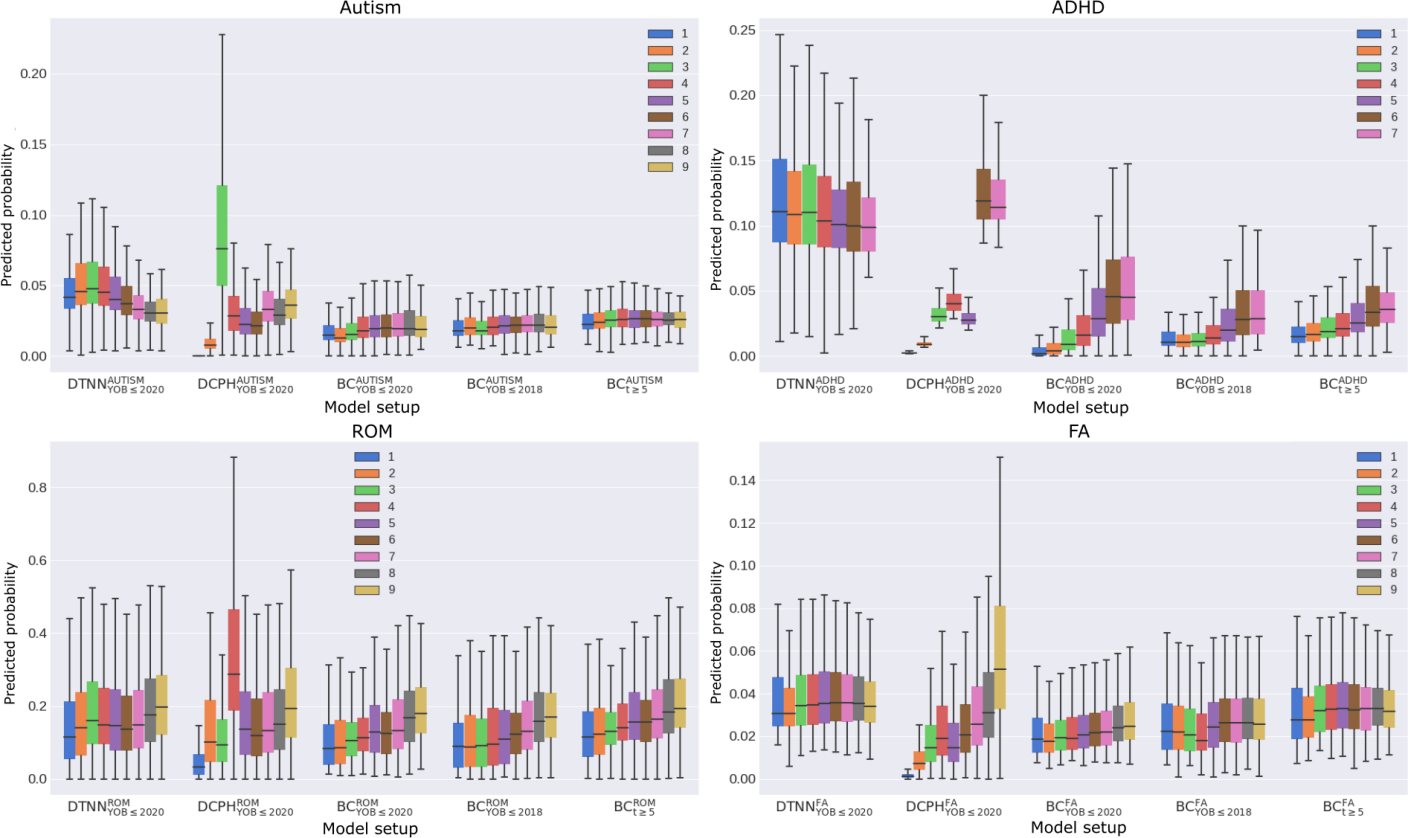
Grouped analysis of predicted probability distributions by follow-up length in years. ADHD: attention-deficit/hyperactivity disorder; BC: binary classification; DCPH: deep Cox proportional hazard; DTNN: discrete-time neural network; FA: food allergy; ROM: recurrent otitis media; t: t denotes follow-up length; YOB: year-of-birth.

There was a positive correlation observed between the predicted probability and follow-up length in all BC models, albeit to a lesser extent in BC_YOB≤2020_ and BC_t≥5_ (Figure 7). A similar trend was apparent in the analysis of the concordance between predicted nonevent probabilities with the observed censoring times (Table 2), with ${\text{BC}}_{\text{YOB≤2020}}^{\text{ADHD}}$ showing the highest concordance index of 0.734. BC predictions appeared to align with the test prevalence (Figures S7-S9 in Multimedia Appendix 1), whereas DTNN and DCPH predictions did not (Figures S5 nd S6 in Multimedia Appendix 1).

**Table 2.** Concordance index by comparing ordered predicted nonevent probabilities of BC^a^ models with observed censoring times.

	Autism	ADHD^b^	ROM^c^	FA^d^
BC_YOB≤2020^e^_	0.581	0.734	0.605	0.558
BC_YOB≤2018_	0.533	0.625	0.605	0.535
BC_t≥5^f^_	0.5	0.605	0.576	0.491

a BC: binary classification.

b ADHD: attention-deficit/hyperactivity disorder.

c ROM: recurrent otitis media.

d FA: food allergy.

e YOB: year-of-birth.

f t denotes follow-up length.

**Figure 8.**
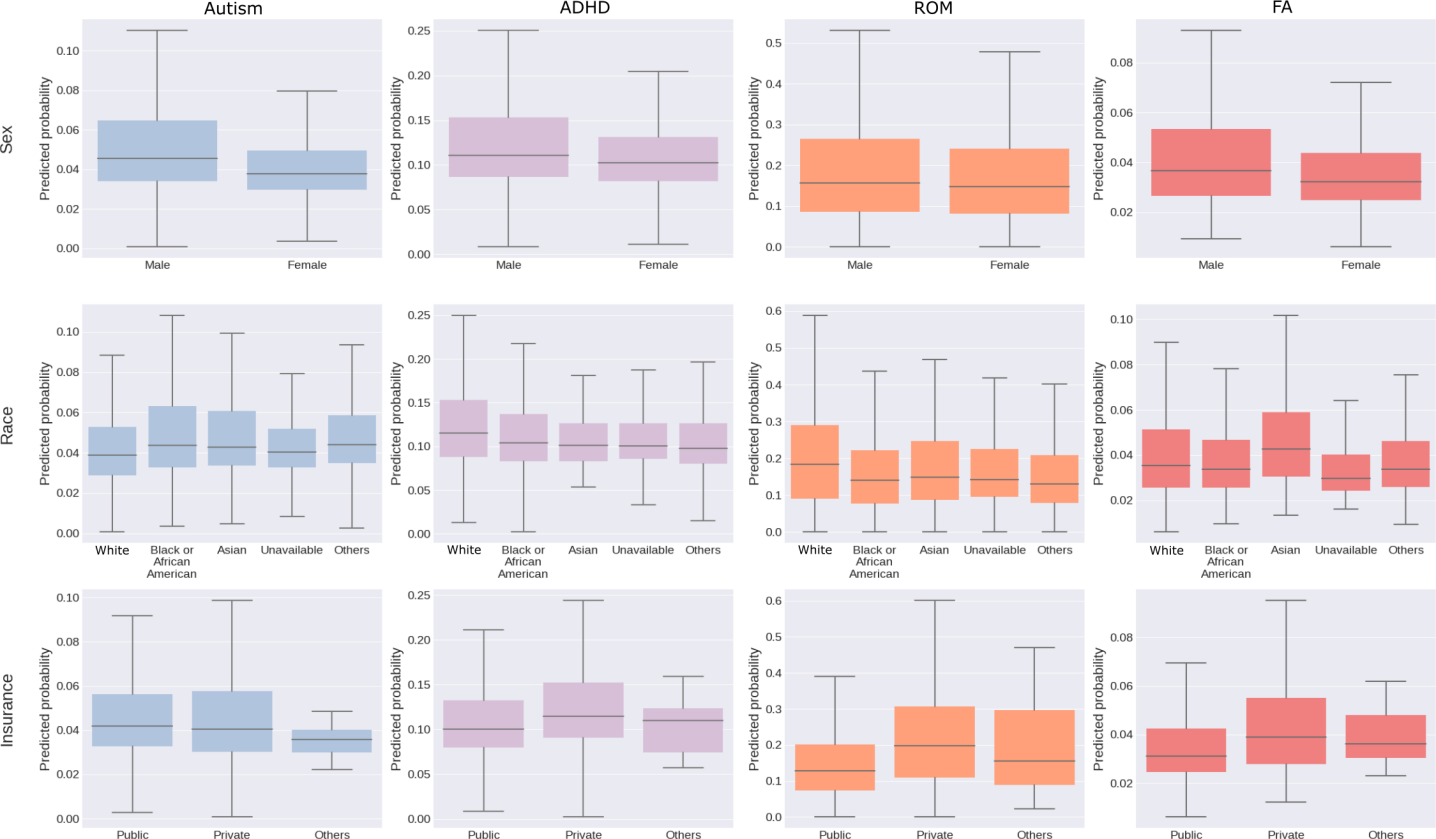
Demographics analysis of probability distributions by DTNN_YOB≤2020_. The subgroups are sex, race, and insurance status. ADHD: attention-deficit/hyperactivity disorder; DTNN: discrete-time neural network; FA: food allergy; ROM: recurrent otitis media; YOB: year-of-birth.

In all 4 conditions, DTNN predicted a greater likelihood of diagnosis for males. Among the racial groups, Asians had the highest predicted probability for autism and FA, while White individuals displayed the highest predicted probability for ADHD and ROM. Regarding insurance status, individuals with private insurance were more likely to be diagnosed with ROM and FA; however, findings for autism and ADHD were equivocal (Figure 8).

The individual results of the subgroup analysis by demographics for each model setup are available in Figures S10-S12 in Multimedia Appendix 1.

## Discussion

### Principal Findings

Our study contributes to the understanding of how right-censoring influences model performance and predicted probabilities over time using EHR data. We highlight inherent limitations of BC in such contexts, even with filtering strategies. Furthermore, our results reinforce the potential of TTE approaches, particularly DTNN, in mitigating bias from the censoring distribution, leading to superior discrimination, calibration, and clinical prevalence prediction.

### Principal Results

First, we demonstrated that BC cannot disentangle the probability of diagnosis and early censoring, even with filtering. The BC models displayed poor AUC_t_ performance, despite achieving high regular AUC scores (Figure 3 and Table S6 in Multimedia Appendix 1). This discrepancy arises because AUC_t_ calculation excludes individuals censored before prediction time *t* whereas regular AUC calculation does not. Thus, the AUC is artificially inflated by “correctly” predicting diagnosed individuals in this subgroup of individuals who were censored early as negative cases. With filtering, BC_YOB≤2018_ and BC_t≥5_ benefitted less, resulting in lower regular AUC scores because more true cases with later diagnoses were excluded.

Spurious positive correlations between the predicted probability and follow-up length imply that BC models were unduly benefitting from early censoring (Figure 7), along with increased concordance between predicted nonevent probabilities and observed censoring times (Table 2). Similarly, these differences were less prominent in BC_YOB≤2020_ and even less in BC_t≥5_, but not completely absent.

This contrast was exacerbated in long-horizon prediction tasks such as ADHD, with the degree of variation corresponding with the tail end of the diagnosis age distributions (Figure 1). ADHD showed the highest proportion of later diagnoses, followed by autism and FA, and the lowest in ROM. These results corroborate observations associating censoring with biased improved outcomes, where hazard ratios fall below 1 compared to complete follow-up and correlate inversely with the proportion of censored cases [27].

Second, we found that TTE models outperformed BC models on all datasets. In diagnoses with longer time horizons, heavy right-censoring leads to many individuals having unknown status, while shorter prediction time horizons tend to have better follow-up. DTNN_YOB≤2020_ and DCPH_YOB≤2020_ achieved comparable or higher AUC_t_ scores in predicting ROM and FA (Figure 3 and Table S6 in Multimedia Appendix 1), suggesting that TTE models matched or surpassed BC models on datasets with less censoring. This superiority is particularly pronounced in autism and ADHD datasets, which experience heavier censoring. The main insight is that TTE models are well-suited to predict clinical outcomes, especially those with prolonged time horizons.

In our semisynthetic ROM censoring experiment, we reproduced the limitations of BC as evidenced by the deterioration in AUC_t_ and regular AUC performance of ${\text{BC}}_{\text{YOB≤2020}}^{\text{ROM, ss}}$ when evaluated on the original dataset (Figure 5 and Table S6 in Multimedia Appendix 1). This result supports our earlier claim that the BC models were underpredicting diagnosed individuals with early censoring. We also demonstrated that ${\text{DTNN}}_{\text{YOB≤2020}}^{\text{ROM, ss}}$ remained well-calibrated and maintained comparable AUC_t_ performance as ${\text{DTNN}}_{\text{YOB≤2020}}^{\text{ROM}}$ (Figure 5), demonstrating the applicability of our TTE approach in situations with partially observed information.

We also examined the impact of BC filtering strategies on OOD years. Specifically, we extended the evaluation to include 2019 and 2020 (Figure 6). Notably, a discernible decline in predicted probabilities was observed for BC_t≥5_ across all clinical conditions, with a slightly more pronounced drop in 2020 compared to 2019. In contrast, predicted probabilities by BC_YOB≤2018_ remained relatively stable during the same OOD years. This suggests that the inclusion of older individuals (ie, born before 2018) with shorter follow-up (ie, <5 years) makes predictions more stable on OOD years. However, including these individuals results in declining predicted probabilities due to early censoring on in-distribution years, as we have previously demonstrated. Moreover, BC_YOB≤2018_ and BC_t≥5_ showed poor calibration for all diagnoses on OOD years (Figure 4), rendering them unsuitable for clinical deployment.

Temporal and demographics trends were poorly represented in BC and DCPH. The probability of diagnosis should remain stable or increase over time due to improved awareness and tools unless specific interventions are implemented. However, BC_YOB≤2020_ exhibited declining predicted probability for all diagnoses because the models assigned lower probability scores to individuals born later, despite the absence of temporal information during learning. Inadvertently, BC predictions follow test prevalence, which also contributes to its poor performance in the demographics subgroup analysis.

The unclear patterns in DCPH models likely result from a violation of the proportional hazards assumption, which is common in practice. For example, varying severity levels in autism and ADHD diagnoses can lead to nonproportionality, where low-likelihood groups initially exhibit delays in hazard before catching up with the high-likelihood groups [28]. By assuming constant hazard rates over time, DCPH models may not fully leverage the complexity of likelihood representations and time-dependent covariate impacts. While excelling in providing generalized representations at a population level (Figure 3 and Figure S4 in Multimedia Appendix 1), our findings suggest inconsistent or inaccurate outcomes in subgroup analyses (Figures6  7, and Figures S10-S12 in Multimedia Appendix 1). DTNN, however, does not assume proportional hazards, enabling better capture of time-dependent covariate influences on survival.

In contrast to the BC and DCPH models, the diagnosis probabilities predicted by the DTNN models (Figures6  8) are in keeping with actual prevalence, reflecting both temporal and demographic trends. For example, autism prevalence increased from 2.24% in 2014 to 2.79% in 2019 [29], with higher rates among males and Black individuals [30]. Our demographics analysis for ADHD also concurs with trends toward increased prevalence in males and White individuals [31]. Note that the reported prevalence in DUHS may exceed nationwide estimates, given its status as a regional hub for neurodevelopmental diagnosis.

Interestingly, for ROM, our DTNN models appear consistent with distinctive temporal patterns including (1) declining prevalence from 2014 to 2017 associated with the availability of postpneumococcal conjugate vaccines [32] and (2) increasing prevalence from 2018 to 2020 amid the COVID-19 pandemic [33]. The DTNN models also accurately predict increased likelihood associated with male sex, White race, lower socioeconomic status [32,34], and private insurance, which reflect health care use disparities [35,36].

Our models suggest stable FA prevalence (~3.4%‐3.5%), adding to mixed data that challenge whether rates have increased (range: 4.8%‐8%) [37]. This discrepancy may arise due to difficulties in estimating true prevalence [38,39] or our stricter diagnostic criteria (*ICD-10* code+IgE-based laboratory test) compared to other studies using surrogate laboratory tests or self-report, which tend to overestimate rates of clinical disease [40-42]. Demographically, our findings corroborate higher FA prevalence among males [43] and Asian and non-Hispanic Black individuals compared to non-Hispanic White individuals [44]. Additionally, our models corroborated the lower FA prevalence reported among children with public insurance [45].

Our findings suggest that TTE models, particularly the DTNN, should be preferred in clinical settings dealing with right censored outcomes. First, the DTNN models outperformed BC models, yielding clinically meaningful discriminatory performance with AUC_t_≥0.7 at early ages across all 4 clinical conditions, supporting earlier diagnoses and timely interventions. Second, the DTNN approach addresses label bias that may lead to underprediction, as evidenced by its superior discrimination, calibration and ability to reflect clinical prevalence. While the modelling approach is arguably more challenging, it avoids the need for complex and often opaque filtering procedures.

### Limitations

Our study has important limitations. First, it is confined to data from DUHS only, which primarily serves a population with a high representation of Black and White individuals. This demographic makeup may limit the generalizability of the results to other health systems with different patient demographics. Second, computable phenotypes are imperfect, as the identification and timing of diagnosis can vary in practice. Third, not all information, including vital signs and laboratory values, was used during the training process. Fourth, we do not include every possible filtering strategy and competing model, which may contribute to the breadth of our findings. Fifth, sex bias may also influence diagnosis trends, with males being more likely to be diagnosed with autism in practice. To the extent that sex affects the distribution of event times, the discrete-time approach can help mitigate this bias, because it does not conflate diagnosis probability with timing unlike BC and DCPH approaches. However, to the extent that sex also influences the probability of diagnosis at any given point, this is not a bias that we can overcome by choice of model alone and will require efforts to change assessment practices. Finally, the constrained size of our dataset prevents us from conducting finer subgroup analyses. For example, we could not explore temporal trends among different demographics, such as instances where autism rates among Black children surpassed those among White children [46]. To address these limitations, we recommend incorporating data from diverse health systems, including a broader range of clinically relevant EHR data, exploring additional filtering strategies, and expanding dataset size to enable more detailed subgroup analyses.

### Conclusion

Machine learning practitioners should acknowledge the inherent limitations of BC on right-censored outcomes and consider TTE approaches, particularly DTNN, in the clinical context. Our study paves the way for future research to identify and optimize models to improve patient outcomes.

## Supplementary material

10.2196/62985 Multimedia Appendix 1 Additional figures and tables.

10.2196/62985 Checklist 1 CREMLS checklist. CREMLS: Consolidated Reporting of Machine Learning Studies.

## Abbreviations

ADHD: attention-deficit/hyperactivity disorder
AP: average precision
AP_t_: time-varying average precision
AUC: area under the receiving operating characteristic curve
AUC_t_: time-varying area under the receiving operating characteristic curve
BC: binary classification
DCPH: deep Cox proportional hazard
DTNN: discrete-time neural network
DUHS: Duke University Health System
EHR: electronic health record
FA: food allergy
*ICD-10*: *International Classification of Diseases, Tenth Revision*
OOD: out-of-distribution
PACE: Protected Analytics Computing Environment
ROM: recurrent otitis media
t: t denotes follow-up length
TTE: time-to-event
YOB: year-of-birth
