# Supplementary material for: Limitations of Binary Classification for Long-Horizon Diagnosis Prediction and Advantages of a Discrete-Time Time-to-Event Approach: Empirical Analysis
Source: JMIR AI. 2025 Mar 27;4:e62985. doi: 10.2196/62985 (PMC12223692; doi:10.2196/62985)
Supplement: Multimedia Appendix 1 [file ai-v4-e62985-s001.docx]

**Figure S1.** Distribution of year of birth for all eligible children in the retrospective cohort.

**
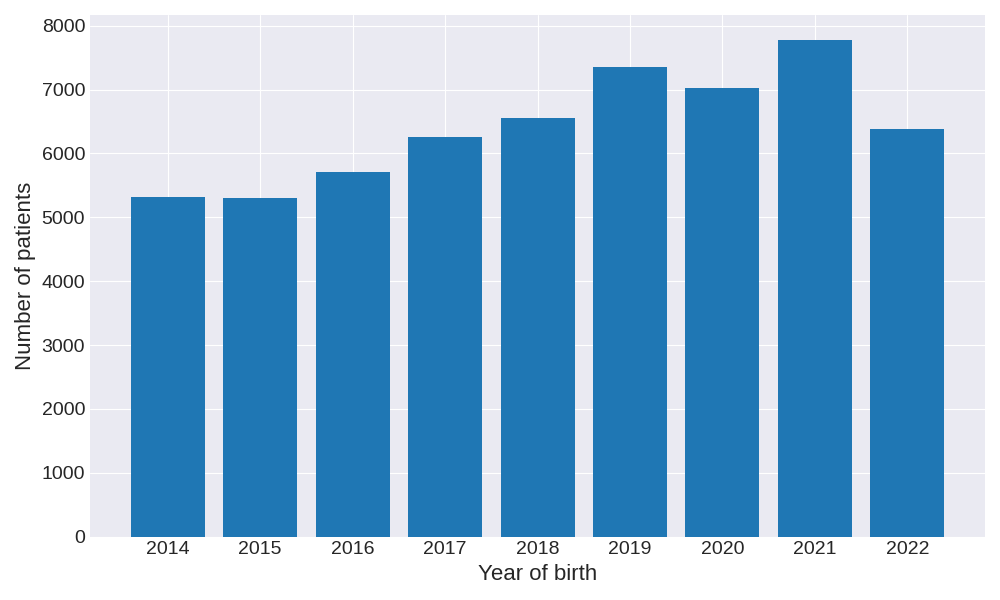
**

**Figure S2.** Distribution of censoring ages in years (upper panel) and months (lower panel). Children with censoring ages before respective diagnosis age cut-offs (marked by the red line) were excluded.

**
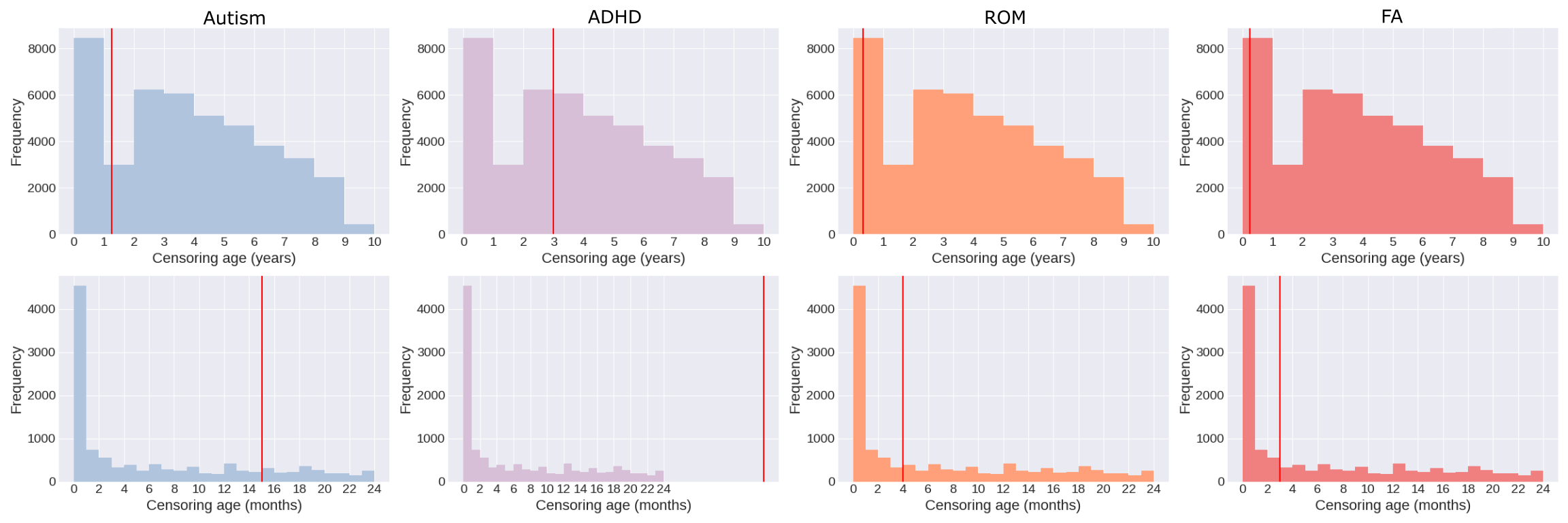
**

**Figure S3.** Comparison of xAP_t_ (solid lines) and regular AP (bar graphs). True prevalence (dashed lines) for each clinical condition was also included.


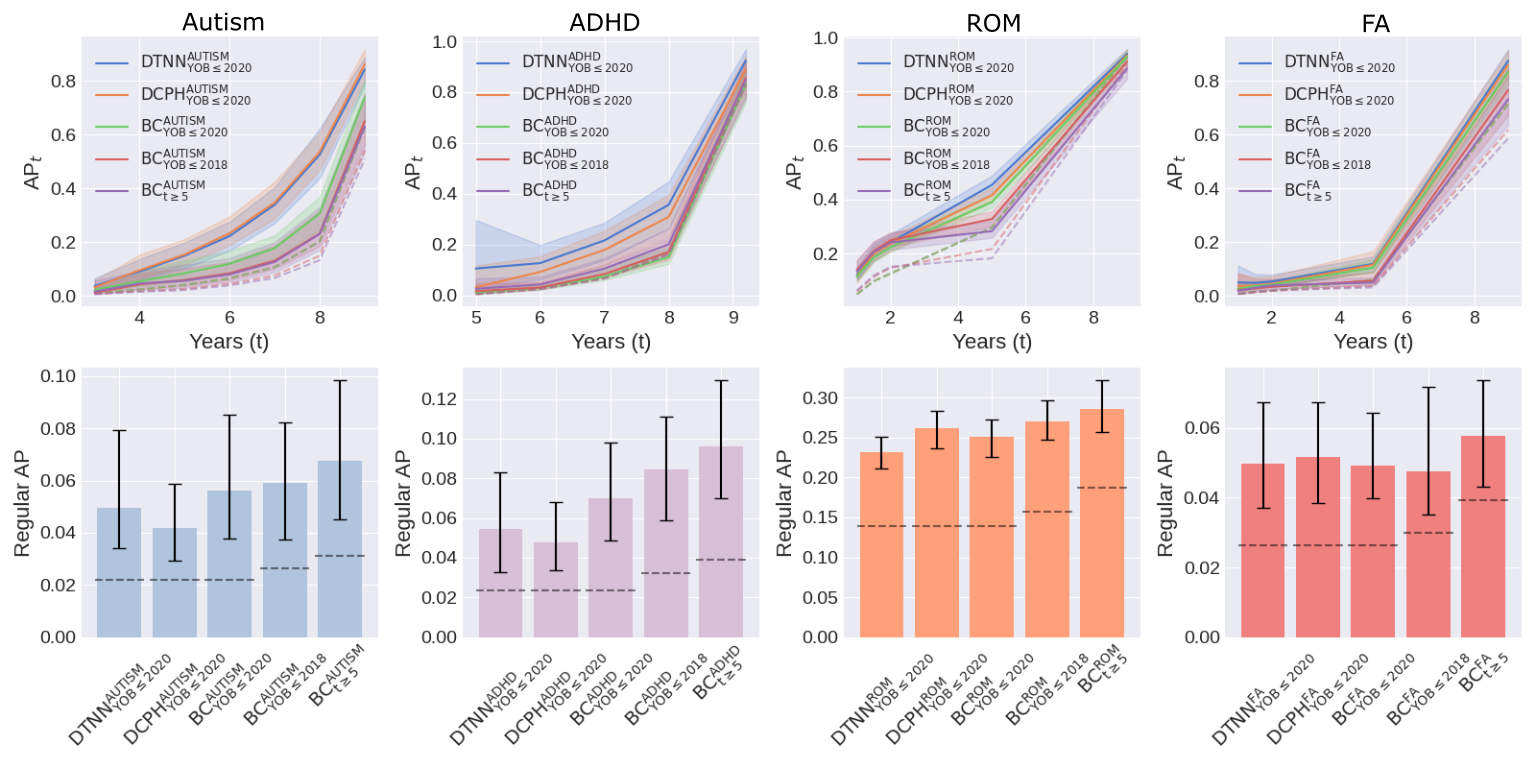


**Figure S4.** Comparison of predicted cumulative probability by TTE models with the Kaplan-Meier curve.

**
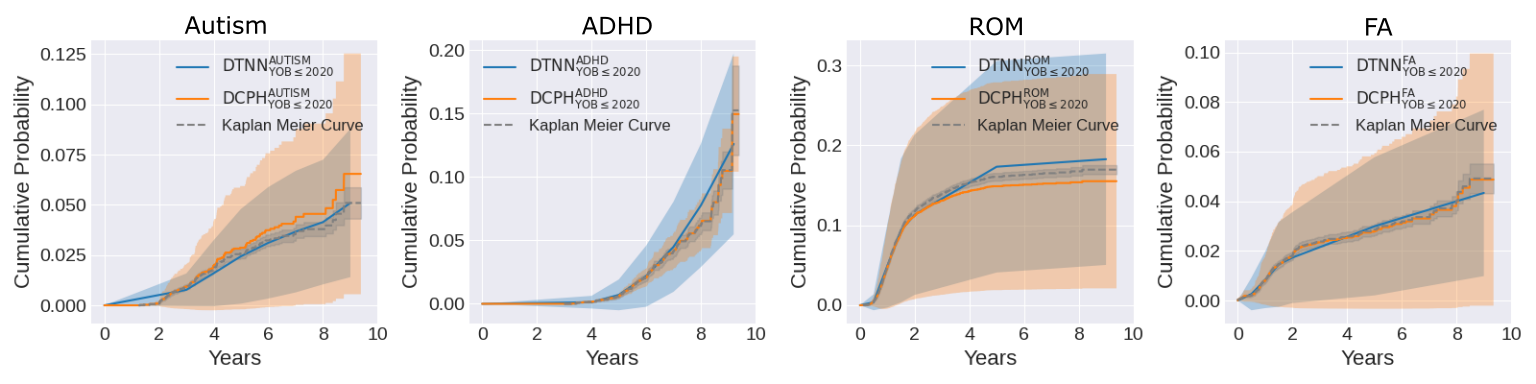
**

**Figure S5.** Analysis of DTNN_YOB≤2020_ predicted probability distributions by year-of-birth.

**
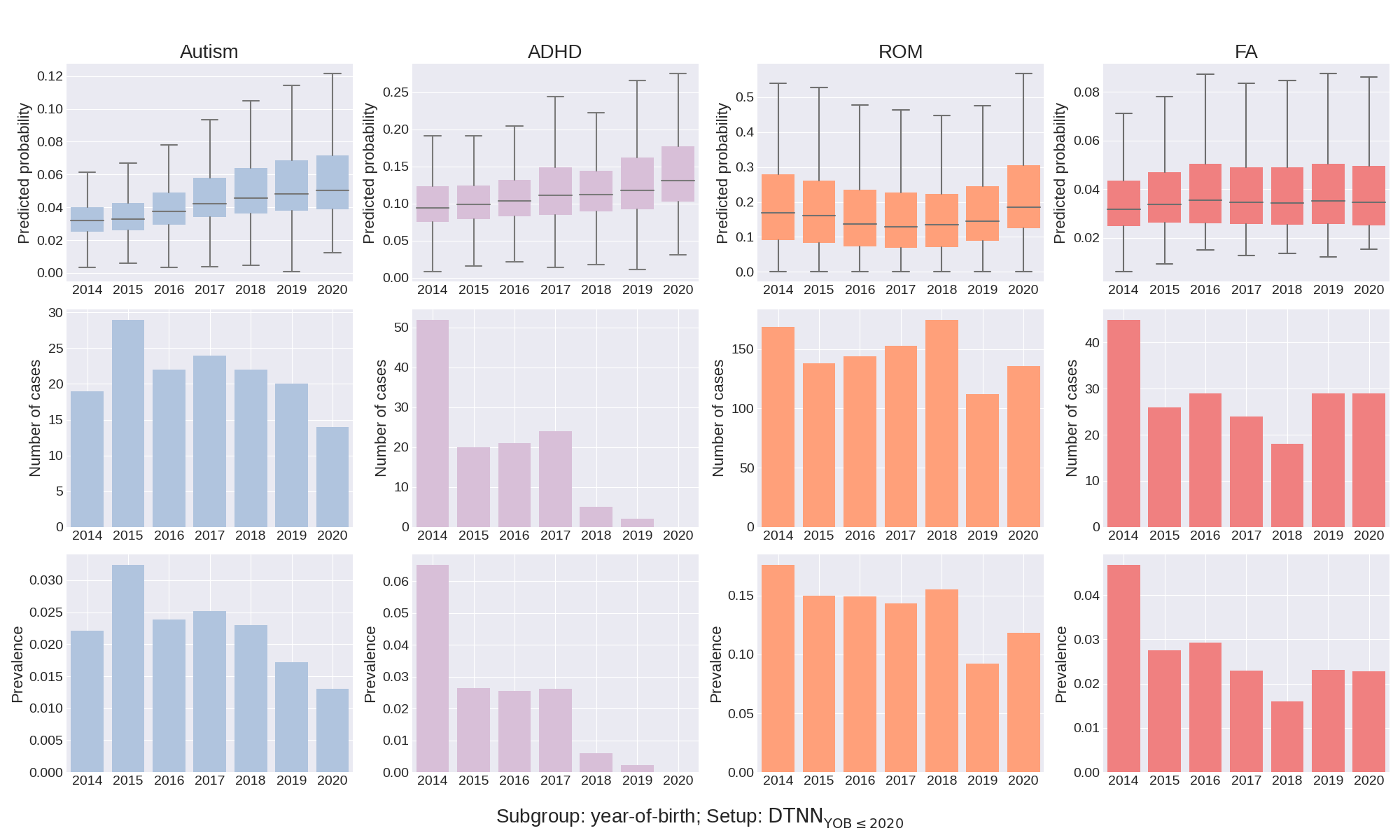
**

**Figure S6.** Analysis of DCPH_YOB≤2020_ predicted probability distributions by year-of-birth.

**
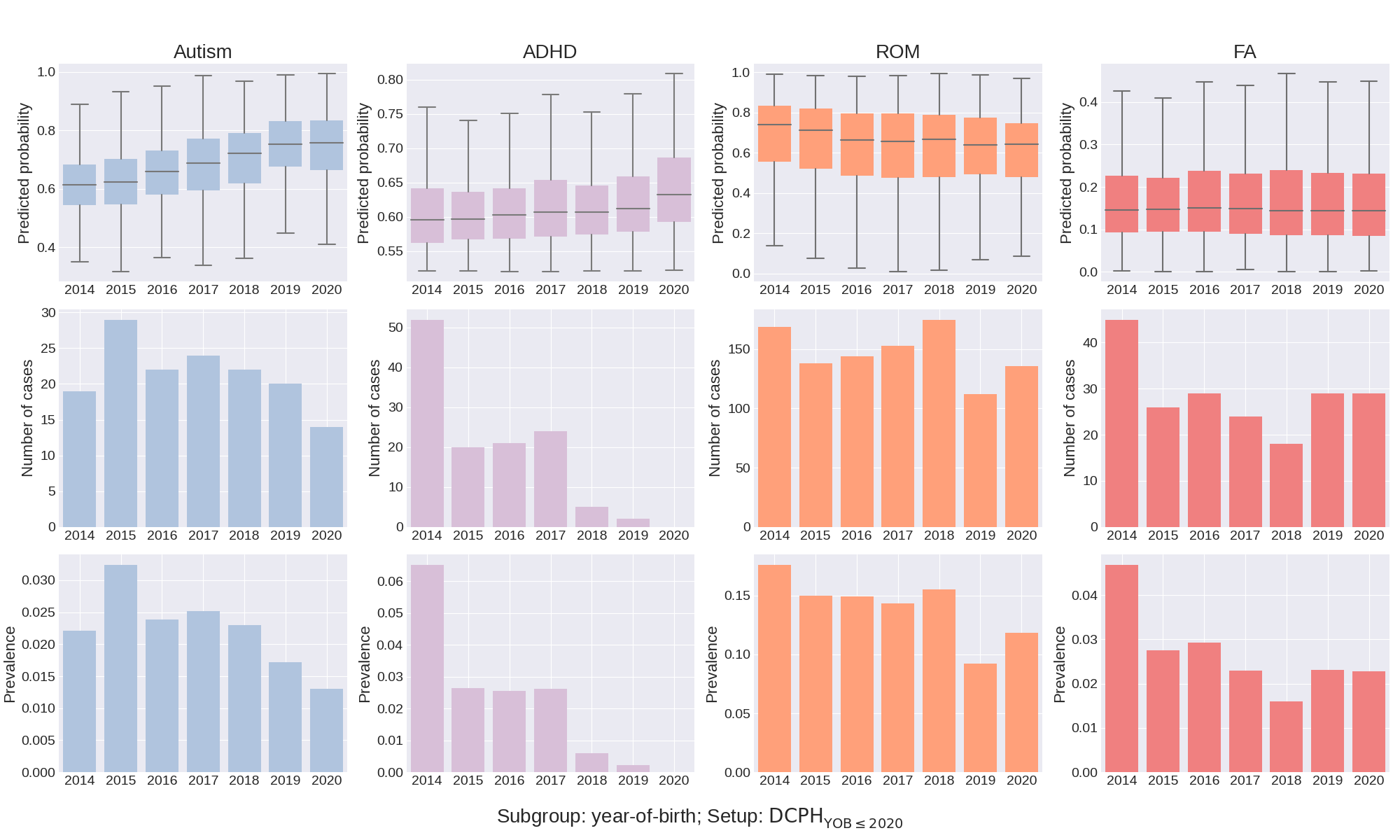
**

**Figure S7.** Analysis of BC_YOB≤2020_ predicted probability distributions by year-of-birth.

**
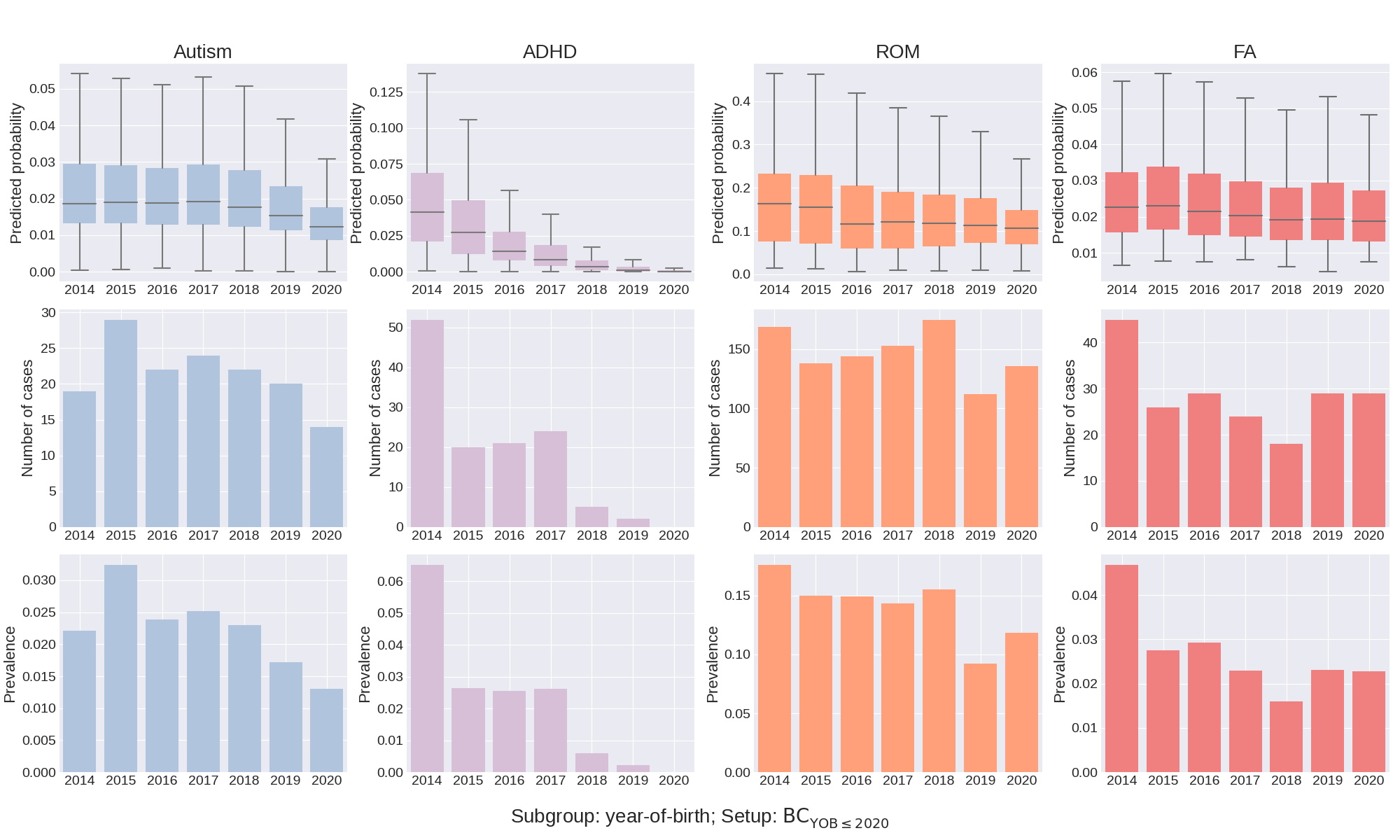
**

**Figure S8.** Analysis of BC_YOB≤2018_ predicted probability distributions by year-of-birth.

**
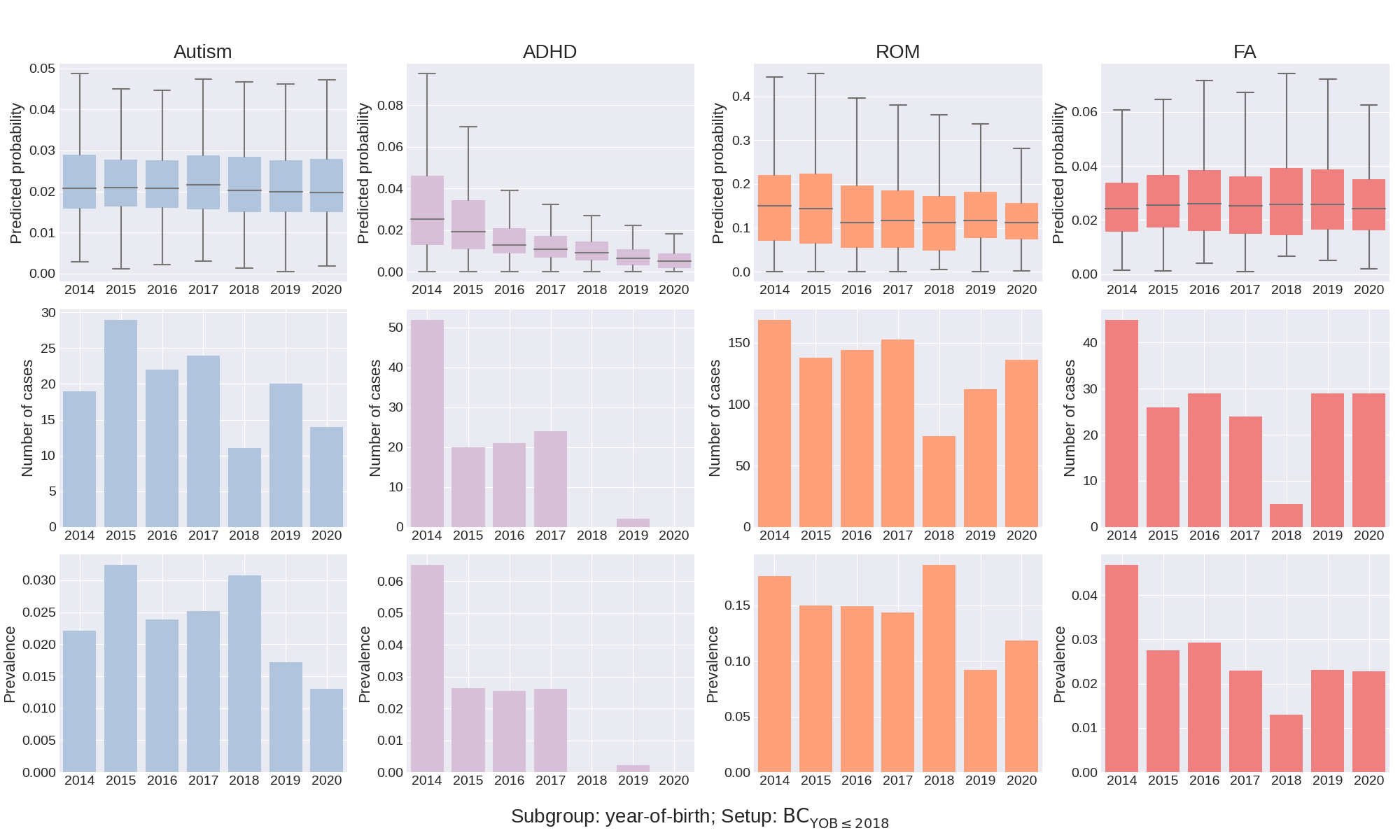
**

**Figure S9.** Analysis of BC_t≥5_ predicted probability distributions by year-of-birth.

**
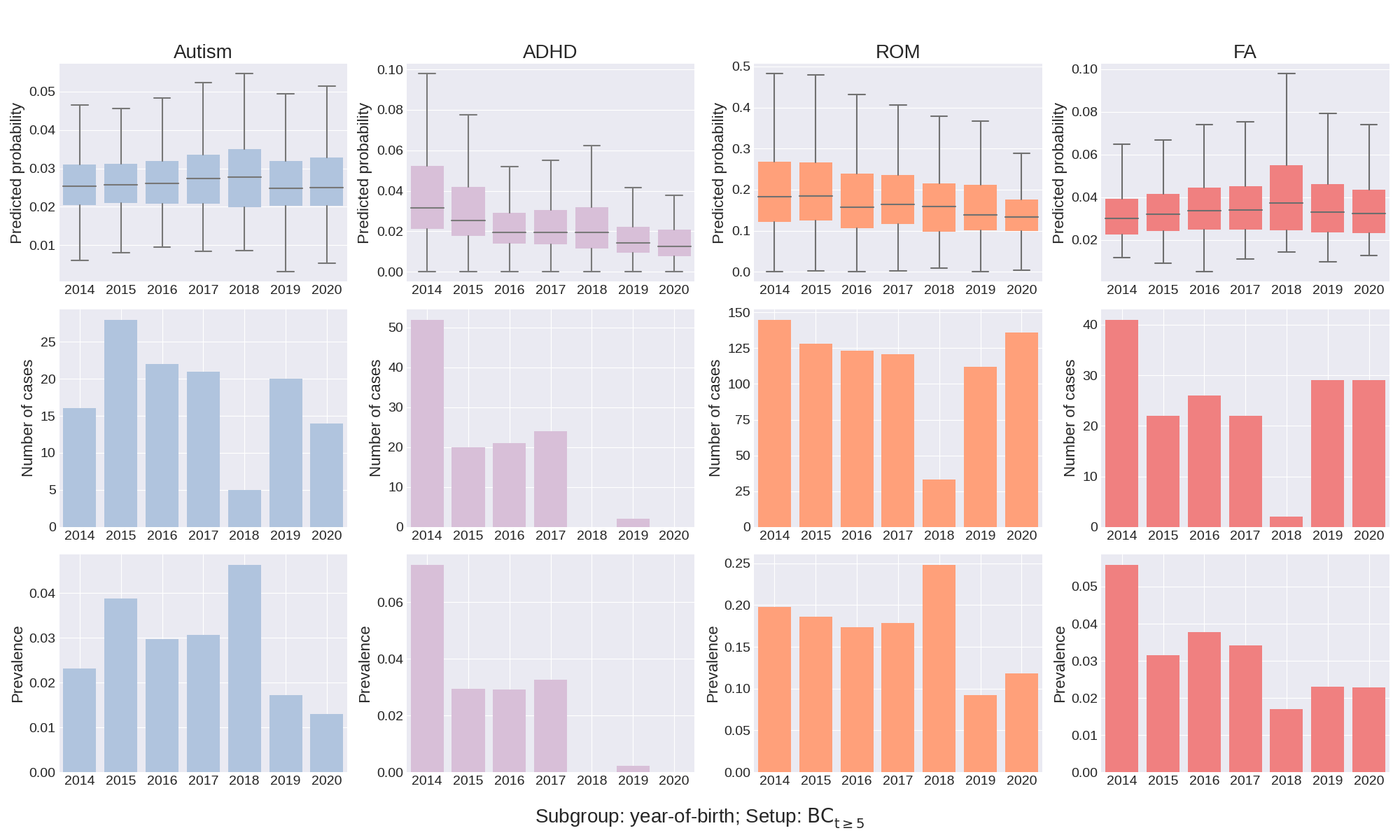
**

**Figure S10.** Grouped analysis of predicted probability distributions by sex.

**
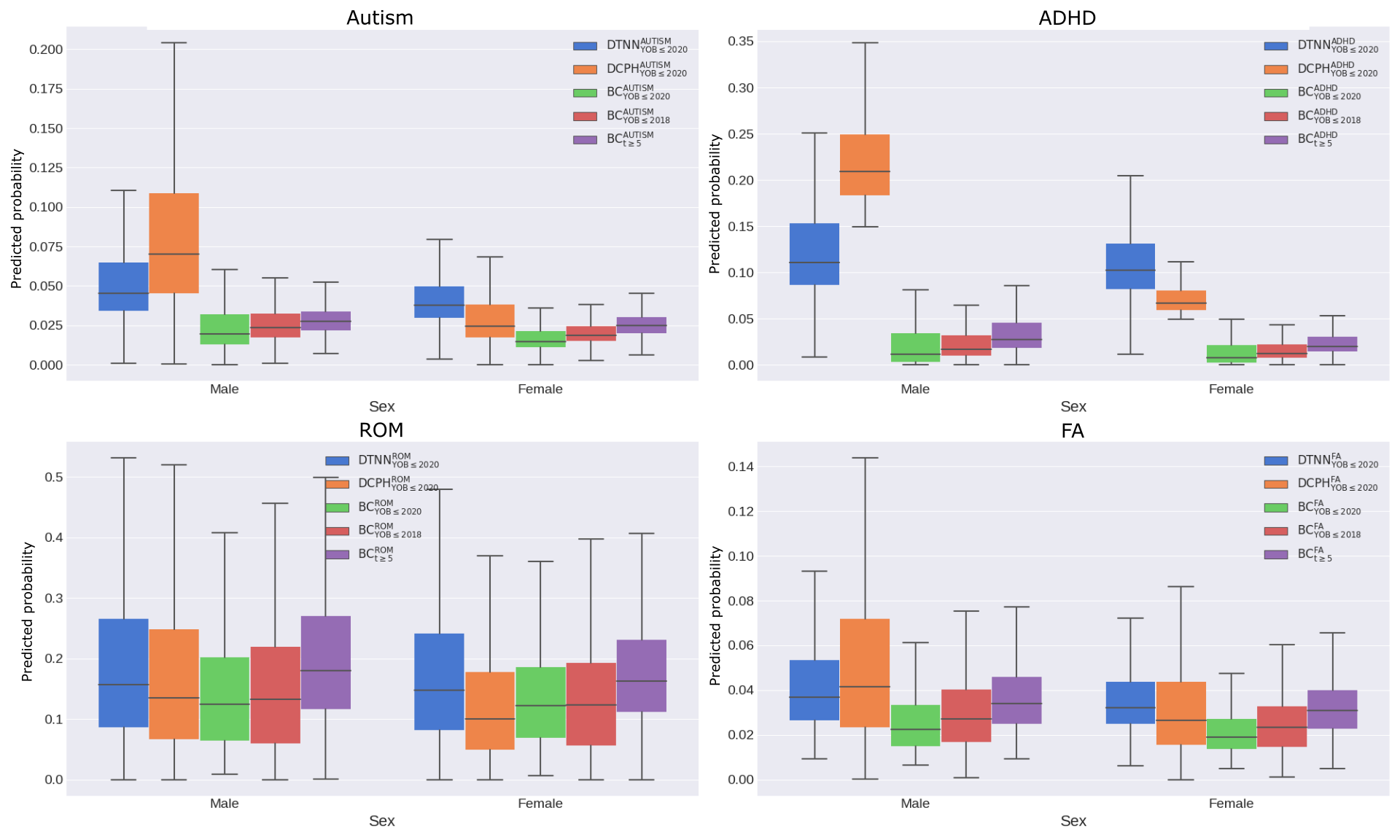
**

**Figure S11.** Grouped analysis of predicted probability distributions by race.

**
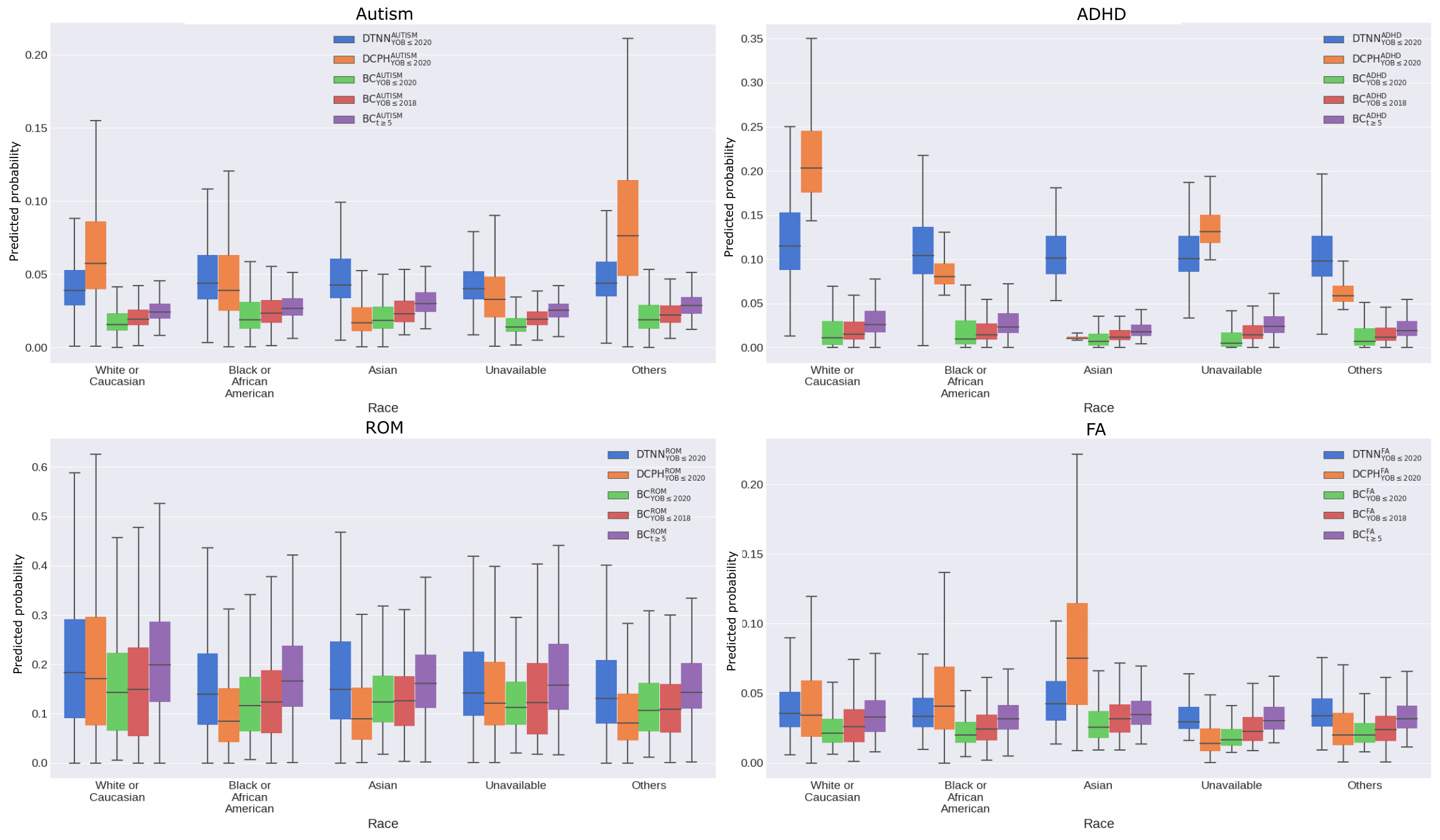
**

**Figure S12.** Grouped analysis of predicted probability distributions by insurance status.

**
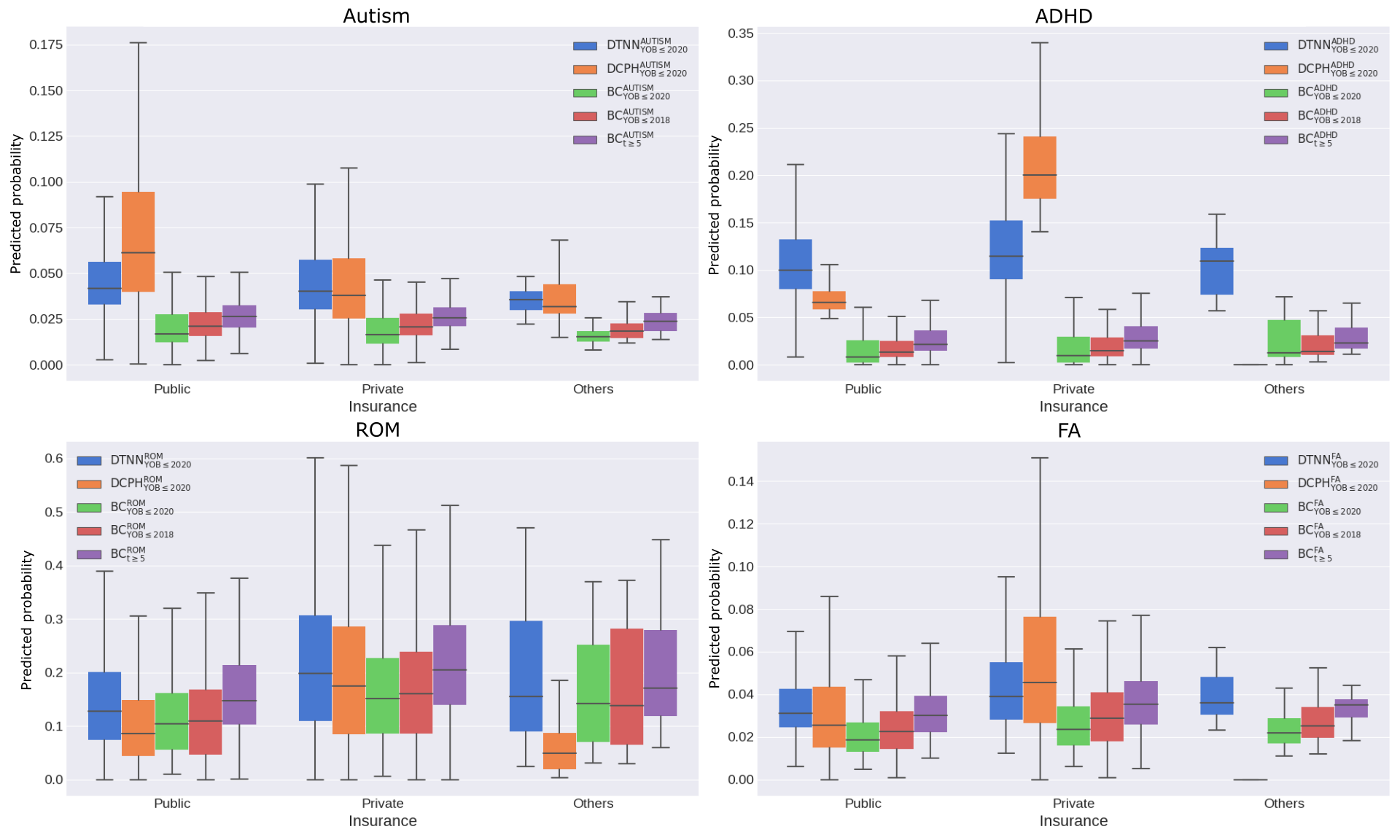
**

**Table S1.** Computable phenotypes criteria and their associated ICD-10 codes.

| **Diagnosis** | **Classification Criteria** | **Associated ICD-10 codes** |
| --- | --- | --- |
| Autism | Meet either of two criteria:   1. 2 or more healthcare encounters associated with an ICD-10 code for autism spectrum disorder 2. at least 1 specialist outpatient encounter associated with an ICD-10 for autism spectrum disorder | F84.0, F84.5, F84.8, F84.9 |
| ADHD | 2 or more healthcare encounters associated with an ICD-10 for ADHD | F90.0, F90.1, F90.2, F90.8, F90.9 |
| Recurrent OM | At least 3 episodes of acute otitis media in a 6-month period or at least 4 within a year. If multiple encounters occur in the same week, it is considered as 1 eligible acute OM encounter.  The requirement for antibiotic prescription has been omitted due to the emphasis on antibiotics in children with uncomplicated acute OM. | H65.0-H65.199, H66-H66.019, H66.4*, H66.9* |
| Food allergy | Meet both criteria:   1. at least 1 health care encounter associated with an ICD-10 for food allergy 2. at least 1 IgE-based lab test (Table S2) | Z91.010, Z91.011, Z91.012, Z91.013, Z91.014, Z91.018 |

**Table S2.** Selected IgE-based lab tests.

| **IgE Allergen** | **Lab Test Code** |
| --- | --- |
| IGE Allergen Chili Pepper | LAB1480 |
| IGE Allergen Chocolate Srm | LAB1144 |
| IGE Allergen Cinnamon | LAB1459 |
| IGE Allergen Clam Serum | LAB633 |
| IGE Allergen Coconut (fruit) | LAB1616 |
| IGE Allergen Cod Fish Serum | LAB602 |
| IGE Allergen Coffee | LAB1161 |
| IGE Allergen Cor a 1 | LAB9890 |
| IGE Allergen Cor a 14 | LAB9893 |
| IGE Allergen Cor a 8 | LAB9891 |
| IGE Allergen Cor a 9 | LAB9892 |
| IGE Allergen Corn Maize | LAB1143 |
| IGE Allergen Crab Serum | LAB1618 |
| IGE Allergen Egg White Components Only | LAB9877 |
| IGE Allergen Egg White Srm | LAB588 |
| IGE Allergen Egg White Srm with Component Reflex | LAB3030 |
| IGE Allergen Egg White Srm with Reflex | LAB9973 |
| IGE Allergen Food Panel | LAB6516 |
| IGE Allergen Food Panel with Component Reflex | LAB3008 |
| IGE Allergen Garlic Serum | LAB1709 |
| IGE Allergen Ginger Spice | LAB1465 |
| IGE Allergen Gluten Serum | LAB1706 |
| IGE Allergen Grain Panel | LAB645 |
| IGE Allergen Halibut Serum | LAB3183 |
| IGE Allergen Hazel Nut Srm with Reflex | LAB9976 |
| IGE Allergen Hazelnut Components Only | LAB9880 |
| IGE Allergen Hazelnut Srm with Component Reflex | LAB3033 |
| IGE Allergen Hazelnut, Srm | LAB614 |
| IGE Allergen Kiwi Fruit | LAB1544 |
| IGE Allergen Lemon Fruit | LAB1606 |
| IGE Allergen Lentil | LAB1585 |
| IGE Allergen Lettuce | LAB1596 |
| IGE Allergen Lobster | LAB642 |
| IGE Allergen Mackerel Serum | LAB3299 |
| IGE Allergen Mango Fruit | LAB1499 |
| IGE Allergen Melons Fruit | LAB1565 |
| IGE Allergen Milk Components Only | LAB9878 |
| IGE Allergen Milk Cow Serum | LAB586 |
| IGE Allergen Milk Cow Serum with Component Reflex | LAB3031 |
| IGE Allergen Milk Cow Serum with Reflex | LAB9974 |
| IGE Allergen Mold Type Cheese | LAB6532 |
| IGE Allergen Mulberry | LAB1588 |
| IGE Allergen Mushroom Serum | LAB1608 |
| IGE Allergen Mustard (Food) | LAB1600 |
| IGE Allergen Oat Grain | LAB643 |
| IGE Allergen Onion Serum | LAB1621 |
| IGE Allergen Orange Fruit | LAB1605 |
| IGE Allergen Oregano Serum | LAB1475 |
| IGE Allergen Ovalbumin | LAB9881 |
| IGE Allergen Ovomucoid | LAB6536 |
| IGE Allergen Oyster Serum | LAB1630 |
| IGE Allergen Papaya Fruit | LAB1592 |
| IGE Allergen Paprika Pepper | LAB1477 |
| IGE Allergen Peach Fruit | LAB1604 |
| IGE Allergen Peanut Components Only | LAB9879 |
| IGE Allergen Peanut Srm | LAB592 |
| IGE Allergen Peanut Srm with Component Reflex | LAB3032 |
| IGE Allergen Peanut Srm with Reflex | LAB9975 |
| IGE Allergen Pear Fruit | LAB6540 |
| IGE Allergen Pecan Nut Srm | LAB1622 |
| IGE Allergen Penicillium Nt | LAB636 |
| IGE Allergen Pepper Black | LAB1479 |
| IGE Allergen Pineapple Fruit | LAB1717 |
| IGE Allergen Pistachio Nut | LAB6543 |
| IGE Allergen Plum Fruit | LAB1581 |
| IGE Allergen Poppy Seed | LAB1482 |
| IGE Allergen Potato Sweet | LAB1578 |
| IGE Allergen Potato White | LAB637 |
| IGE Allergen Rye Grain | LAB1623 |
| IGE Allergen Salmon Serum | LAB1624 |
| IGE Allergen Scallop | LAB1708 |
| IGE Allergen Shrimp Serum | LAB617 |
| IGE Allergen Squash | LAB1710 |
| IGE Allergen Squid | LAB6548 |
| IGE Allergen Strawberry | LAB609 |
| IGE Allergen Sunflower Seed | LAB1597 |
| IGE Allergen Trout | LAB1571 |
| IGE Allergen Trout | LAB3181 |
| IGE Allergen Turkey Meat | LAB1627 |
| IGE Allergen Vanilla Serum | LAB1491 |
| IGE Allergen Walley Pike Serum | LAB3182 |
| IGE Allergen Walnut Nut | LAB604 |
| IgE Allergen Beta-Lactoglobulin | LAB9883 |
| IgE Ab Allergen Nutmeg Spice | LAB1472 |
| IgE Allergen Cheddar Cheese | LAB1521 |
| IgE Allergen Fennel Greek | LAB6561 |
| IgE Allergen Grapefruit | LAB6564 |
| IgE Allergen Grapes | LAB1584 |
| IgE Allergen Green Pea | LAB622 |
| IgE Allergen Green Pepper | LAB1481 |
| IgE Allergen Halibut | LAB1562 |
| IgE Allergen Pine Nut Serum | LAB6565 |
| IgE Allergen Pork | LAB1607 |
| IgE Allergen Rice Grain | LAB624 |
| IgE Allergen Seafood Panel | LAB596 |
| IgE Allergen Sesame Seed | LAB1625 |
| IgE Allergen Soybean Serum | LAB587 |
| IgE Allergen Tea Serum | LAB1626 |
| IgE Allergen Tilapia | LAB6568 |
| IgE Allergen Tomato | LAB625 |
| IgE Allergen Tuna Serum | LAB640 |
| IgE Allergen Wheat Grain | LAB590 |
| IgE Allergen Whey Serum | LAB1522 |
| IgE Food Allergy Profile | LAB1156 |
| IgE Food Allergy Profile with Component Reflex | LAB3010 |

**Table S3.** Parameter values used during Word2Vec model training. Note that some individuals in the pretraining cohort could be reused in the train set of the task-specific cohort, however there would be no overlap with individuals in the validation and test sets of the task-specific cohorts to prevent data leakage.

| **Parameter** | **Value** |
| --- | --- |
| *min_count* | 0 |
| *Window* | 3 |
| *vector_size* | 256 |
| *Sample* | 6e-5 |
| *Alpha* | 0.01 |
| *min_alpha* | 1e-4 |
| *Negative* | 20 |

**Table S4.** Defined parameters for each clinical condition. The parameters include diagnosis age cut-offs and bin boundaries used for TTE analysis.

|  | **Autism** | **ADHD** | **Recurrent OM** | **Food Allergy** |
| --- | --- | --- | --- | --- |
| **Diagnosis age cut-offs (years)** | 1.25 | 3.0 | 0.333 | 0.25 |
| **Bin Boundaries (years)** | [0, 3, 4, 5, 6, 7, 8, 9] | [0, 4, 5, 6, 7, 8, 9.2] | [0, 0.5, 1, 1.5, 2, 5, 9] | [0, 0.5, 1, 1.5, 2, 5, 9] |

**Table S5.** Summary of optimal hyperparameters obtained through grid search. The hyperparameters for $\text{DTNN}_{\text{YOB≤2020}}^{\text{ROM, ss}}$ and $\text{BC}_{\text{YOB≤2020}}^{\text{ROM, ss}}$ are enclosed in parenthesis.

|  | | **Autism** | **ADHD** | **Recurrent OM** | **Food Allergy** |
| --- | --- | --- | --- | --- | --- |
| **DTNN_YOB≤2020_** | **Learning Rate** | 1e-4 | 1e-2 | 1e-3 (1e-2) | 1e-4 |
|  | **Weight Decay** | 1e-6 | 1e-5 | 1e-6 (1e-5) | 1e-7 |
| **DCPH_YOB≤2020_** | **Learning Rate** | 1e-2 | 1e-2 | 1e-3 | 1e-3 |
|  | **Weight Decay** | 1e-6 | 1e-5 | 1e-7 | 1e-5 |
| **BC_YOB≤2020_** | **Learning Rate** | 1e-4 | 1e-2 | 1e-2 (1e-3) | 1e-2 |
|  | **Weight Decay** | 1e-7 | 1e-6 | 1e-5 (1e-5) | 1e-5 |
| **BC_YOB≤2018_** | **Learning Rate** | 1e-4 | 1e-2 | 1e-3 | 1e-4 |
|  | **Weight Decay** | 1e-6 | 1e-5 | 1e-7 | 1e-7 |
| **BC_t≥5_** | **Learning Rate** | 1e-2 | 1e-2 | 1e-3 | 1e-4 |
|  | **Weight Decay** | 1e-5 | 1e-6 | 1e-5 | 1e-6 |

**Table S6.** Regular and time-varying AUC for each model setup. The values for $\text{DTNN}_{\text{YOB≤2020}}^{\text{ROM, ss}}$ and $\text{BC}_{\text{YOB≤2020}}^{\text{ROM, ss}}$ are enclosed in parenthesis.

|  | | **DTNN_YOB≤2020_** | **DCPH_YOB≤2020_** | **BC_YOB≤2020_** | **BC_YOB≤2018_** | **BC_t≥5_** |
| --- | --- | --- | --- | --- | --- | --- |
| **Autism** | **AUC** | 0.594 | 0.606 | 0.656 | 0.635 | 0.622 |
|  | **xAUC_3_** | 0.707 | 0.694 | 0.617 | 0.615 | 0.647 |
|  | **xAUC_4_** | 0.708 | 0.723 | 0.626 | 0.66 | 0.686 |
|  | **xAUC_5_** | 0.703 | 0.722 | 0.603 | 0.632 | 0.657 |
|  | **xAUC_6_** | 0.712 | 0.729 | 0.601 | 0.622 | 0.64 |
|  | **xAUC_7_** | 0.747 | 0.757 | 0.603 | 0.622 | 0.646 |
|  | **xAUC_8_** | 0.765 | 0.774 | 0.599 | 0.62 | 0.65 |
|  | **xAUC_9_** | 0.757 | 0.781 | 0.607 | 0.63 | 0.634 |
| **ADHD** | **AUC** | 0.638 | 0.641 | 0.753 | 0.708 | 0.694 |
|  | **xAUC_4_** | 0.893 | 0.835 | 0.475 | 0.888 | 0.912 |
|  | **xAUC_5_** | 0.696 | 0.689 | 0.541 | 0.676 | 0.726 |
|  | **xAUC_6_** | 0.703 | 0.658 | 0.475 | 0.529 | 0.605 |
|  | **xAUC_7_** | 0.721 | 0.678 | 0.473 | 0.553 | 0.608 |
|  | **xAUC_8_** | 0.681 | 0.635 | 0.408 | 0.497 | 0.544 |
|  | **xAUC_9.2_** | 0.716 | 0.601 | 0.423 | 0.533 | 0.543 |
| **Recurrent OM** | **AUC** | 0.663 (0.66) | 0.683 | 0.686 (0.635) | 0.68 | 0.652 |
|  | **xAUC_0.5_** | 0.623 (0.696) | 0.659 | 0.669 (0.693) | 0.662 | 0.636 |
|  | **xAUC_1_** | 0.721 (0.707) | 0.714 | 0.726 (0.688) | 0.733 | 0.704 |
|  | **xAUC_1.5_** | 0.692 (0.678) | 0.688 | 0.688 (0.65) | 0.686 | 0.676 |
|  | **xAUC_2_** | 0.681 (0.645) | 0.679 | 0.679 (0.642) | 0.676 | 0.662 |
|  | **xAUC_5_** | 0.662 (0.612) | 0.636 | 0.623 (0.486) | 0.65 | 0.655 |
|  | **xAUC_9_** | 0.548 (0.535) | 0.537 | 0.518 (0.34) | 0.558 | 0.539 |
| **Food Allergy** | **AUC** | 0.645 | 0.653 | 0.668 | 0.605 | 0.597 |
|  | **xAUC_0.5_** | 0.725 | 0.619 | 0.722 | 0.345 | 0.466 |
|  | **xAUC_1_** | 0.738 | 0.706 | 0.767 | 0.614 | 0.667 |
|  | **xAUC_1.5_** | 0.687 | 0.671 | 0.708 | 0.632 | 0.66 |
|  | **xAUC_2_** | 0.679 | 0.675 | 0.699 | 0.643 | 0.66 |
|  | **xAUC_5_** | 0.658 | 0.65 | 0.643 | 0.603 | 0.627 |
|  | **xAUC_9_** | 0.732 | 0.69 | 0.658 | 0.656 | 0.661 |

**Table S7.** Regular and time-varying AP for each model setup. The values for $\text{DTNN}_{\text{YOB≤2020}}^{\text{ROM, ss}}$ and $\text{BC}_{\text{YOB≤2020}}^{\text{ROM, ss}}$ are enclosed in parenthesis.

|  | | **DTNN_YOB≤2020_** | **DCPH_YOB≤2020_** | **BC_YOB≤2020_** | **BC_YOB≤2018_** | **BC_t≥5_** |
| --- | --- | --- | --- | --- | --- | --- |
| **Autism** | **AP** | 0.05 | 0.042 | 0.056 | 0.059 | 0.068 |
|  | **xAP_3_** | 0.037 | 0.032 | 0.023 | 0.012 | 0.016 |
|  | **xAP_4_** | 0.091 | 0.095 | 0.054 | 0.042 | 0.046 |
|  | **xAP_5_** | 0.15 | 0.154 | 0.084 | 0.059 | 0.056 |
|  | **xAP_6_** | 0.223 | 0.233 | 0.119 | 0.084 | 0.08 |
|  | **xAP_7_** | 0.339 | 0.347 | 0.177 | 0.131 | 0.126 |
|  | **xAP_8_** | 0.525 | 0.535 | 0.306 | 0.232 | 0.23 |
|  | **xAP_9_** | 0.842 | 0.862 | 0.738 | 0.65 | 0.63 |
| **ADHD** | **AP** | 0.055 | 0.048 | 0.07 | 0.084 | 0.096 |
|  | **xAP_4_** | 0.056 | 0.034 | 0.003 | 0.007 | 0.009 |
|  | **xAP_5_** | 0.106 | 0.033 | 0.012 | 0.017 | 0.027 |
|  | **xAP_6_** | 0.127 | 0.093 | 0.03 | 0.032 | 0.044 |
|  | **xAP_7_** | 0.217 | 0.179 | 0.075 | 0.085 | 0.106 |
|  | **xAP_8_** | 0.357 | 0.309 | 0.151 | 0.172 | 0.201 |
|  | **xAP_9.2_** | 0.925 | 0.89 | 0.83 | 0.857 | 0.849 |
| **Recurrent OM** | **AP** | 0.232 (0.22) | 0.262 | 0.252 (0.221) | 0.27 | 0.286 |
|  | **xAP_0.5_** | 0.015 (0.013) | 0.012 | 0.015 (0.013) | 0.024 | 0.016 |
|  | **xAP_1_** | 0.120 (0.111) | 0.117 | 0.116 (0.107) | 0.14 | 0.134 |
|  | **xAP_1.5_** | 0.203 (0.182) | 0.2 | 0.186 (0.173) | 0.209 | 0.206 |
|  | **xAP_2_** | 0.241 (0.212) | 0.237 | 0.224 (0.207) | 0.249 | 0.241 |
|  | **xAP_5_** | 0.455 (0.393) | 0.416 | 0.391 (0.307) | 0.328 | 0.283 |
|  | **xAP_9_** | 0.939 (0.935) | 0.934 | 0.93 (0.895) | 0.911 | 0.886 |
| **Food Allergy** | **AP** | 0.05 | 0.052 | 0.049 | 0.048 | 0.058 |
|  | **xAP_0.5_** | 0.011 | 0.018 | 0.008 | 0.001 | 0.002 |
|  | **xAP_1_** | 0.051 | 0.036 | 0.028 | 0.023 | 0.02 |
|  | **xAP_1.5_** | 0.049 | 0.039 | 0.037 | 0.028 | 0.031 |
|  | **xAP_2_** | 0.054 | 0.046 | 0.042 | 0.034 | 0.039 |
|  | **xAP_5_** | 0.119 | 0.116 | 0.105 | 0.057 | 0.05 |
|  | **xAP_9_** | 0.874 | 0.859 | 0.832 | 0.766 | 0.731 |

**Table S8.** Concordance index by comparing ordered predicted event probabilities with observed event times.

|  | **Autism** | **ADHD** | **Recurrent OM** | **Food Allergy** |
| --- | --- | --- | --- | --- |
| **DTNN_YOB≤2020_** | 0.656 | 0.682 | 0.652 | 0.658 |
| **DCPH_YOB≤2020_** | 0.667 | 0.657 | 0.667 | 0.657 |
| **BC_YOB≤2020_** | 0.629 | 0.558 | 0.669 | 0.68 |
| **BC_YOB≤2018_** | 0.631 | 0.614 | 0.664 | 0.61 |
| **BC_t≥5_** | 0.635 | 0.645 | 0.642 | 0.61 |
