# Supplementary material for: Limitations of Binary Classification for Long-Horizon Diagnosis Prediction and Advantages of a Discrete-Time Time-to-Event Approach: Empirical Analysis
Source: JMIR AI. 2025 Mar 27;4:e62985. doi: 10.2196/62985 (PMC12223692; doi:10.2196/62985)
Supplement: Checklist 1 [file ai-v4-e62985-s002.docx]

**Study details**

| (1.1) The medical or clinical task of interest | We examined the advantages of the DTNN approach compared to BC and DCPH across four long-horizon, EHR-based event prediction tasks. |
| --- | --- |
| (1.2) The research question | This empirical analysis compared different model approaches – BC with filtering strategies, DCPH and DTNN – to characterize the inherent limitations of BC for long-horizon diagnosis from EHR and quantify the benefits of the DTNN approach. |
| (1.3) Current medical or clinical practice | For clinical diagnoses, we used computable phenotypes previously established within DUHS or formulated in consultation with clinicians. The classification criteria are provided in Tables S1 & S2. |
| (1.4) The known predictors and confounders of what is being predicted or diagnosed | Input variables included timestamped sequences of DUHS EHR events, such as ICD-10 diagnoses, RxNorm medications, CPT procedures, and LOINC lab results. Output variables, as detailed in Table 2, were binned probabilities for DTNN, log hazard ratios for DCPH, and log odds for BC, all ultimately converted to probabilities for comparison. |
| (1.5) The overall study design | Please refer to the “Experimental Setup” section, where we described the setup of various modeling approaches for predicting each of the four clinical conditions. This includes filtering strategies, feature extraction based on predefined condition-specific prediction ages, and the train-validation-test data partitioning. |
| (1.6) The medical institutional settings | Please refer to the “Cohort Identification” section. Analyses were based on inpatient and outpatient encounters within the DUHS, a large academic medical center based in Durham, North Carolina. DUHS provides care to approximately 85% of children in Durham and surrounding Durham County, which has a diverse population with varying demographic and socioeconomic status. |
| (1.7) The target patient population | The target patient population are the children within DUHS EHR. The study inclusion criteria were (1) date of birth between January 1, 2014 and October 29, 2022; and (2) ≥ 1 visit within the DUHS before 30 days old. |
| (1.8) The intended use of the ML model | We demonstrated that the DTNN models had superior and clinically meaningful performance in predicting conditions, especially long-horizon diagnoses, using EHR data. This approach can be integrated into clinical practice for earlier diagnoses and timely interventions, ultimately supporting patient outcomes. We are transparent in our design and have provided the code to implement the models. |
| (1.9) Existing model performance | We compared the performance of the DTNN with established model approaches, such as BC with various filtering strategies and DCPH, which is widely recognised as a benchmark for TTE clinical prediction tasks. |
| (1.10) Ethical and other regulatory approvals obtained | Please refer to the “Institutional Review and Informed Consent” section. All study procedures were approved by the Duke Health Institutional Review Board and comply with institutional policies and federal regulations. Analyses were executed within the Duke Protected Analytics Computing Environment (PACE), a highly protected virtual network space designed for protected health information. |

**The data**

| (2.1) Inclusion or exclusion criteria for the patient cohort | Please refer to the “Cohort Identification” section. The inclusion criteria for the patient cohort were: (1) date of birth between January 1, 2014, and October 29, 2022; and (2) at least one visit to DUHS before 30 days of age. Additionally, we included patients meeting the classification criteria for each computable phenotype, as detailed in Tables S1 & S2. |
| --- | --- |
| (2.2) Methods of data collection | The data for this study were sourced from the DUHS EHR. We described the methods and types of data used in the “Model Development” section. In brief, this includes timestamped patient histories such as ICD-10 codes, medications, procedures, and lab results. A diagram outlining the input-output parsing process is shown in Figure 2. |
| (2.3) Bias introduced due to the method of data collection used | Biases in our study, including those arising from the demographic composition of the DUHS dataset, which may limit generalizability, and sex bias in diagnosis trends (e.g., autism), have been acknowledged in the “Limitations” section. The most significant bias inherent in EHR data is right-censoring, which we have comprehensively addressed in this paper. |
| (2.4) Data characteristics | Descriptive statistics for key demographic (sex, race, insurance status) across clinical conditions (autism, ADHD, recurrent OM, and food allergy) are shown in Table 1, with statistical tests for group differences. |
| (2.5) Methods of data transformation and preprocessing applied | Please refer to the “Experimental Setup” and “Model Development” sections for detailed data preprocessing steps, including filtering by birth year, follow-up duration, and the use of pretrained medical concept embeddings. Implementation codes are available online on GitHub. |
| (2.6) Known quality issues with the data | Data quality issues, such as the exclusion of outliers like an individual diagnosed with autism within the first month of life, were carefully managed during preprocessing. |
| (2.7) Sample size calculation | We are utilizing electronic health records from 57,701 individuals, which is orders of magnitude larger than the minimum sample size required for effective model development. |
| (2.8) Data availability | Due to the sensitive nature of the data involved, which includes EHR, we are unable to provide access to the dataset in accordance with privacy regulations and ethical considerations. |

**Methodology**

| (3.1) Strategies for handling missing data | Our approach views electronic health records as a sequence of events (e.g., diagnosis codes, procedure codes), each of which is mapped to a corresponding dense vector embedding. We do not assume that any particular predictor or code is present, therefore there is no need to impute missing values. |
| --- | --- |
| (3.2) Strategies for addressing class imbalance | We did not adjust for class imbalance due to (a) adequate representation of the various positive classes (i.e., diagnoses of interest), and (b) concerns about the effects of class balancing on model calibration. |
| (3.3) Strategies for reducing dimensionality of data | We reduced dimensionality using embeddings with pre-trained weights and applied global mean pooling to obtain fixed-length vector representations. For further details, please refer to the “Encoder Architecture” section. |
| (3.4) Strategies for handling outliers | We excluded outliers, including the individual diagnosed with autism within the first month of life, and those diagnosed or censored before predefined, condition-specific prediction ages: 15 months for autism, 3 years for ADHD, 4 months for recurrent OM, and 3 months for food allergy (Figure 1). |
| (3.5) Strategies for data augmentation | No data augmentation steps were applied. |
| (3.6) Strategies for model pretraining | Word2Vec was used to pretrain medical concept embeddings on DUHS EHR event sequences, capturing contextual relationships between diagnosis codes, medications, procedures, and labs. Please refer to the “Pretraining Medical Concept Embeddings” section for details. Implementation code is also provided. |
| (3.7) The rationale for selecting the ML algorithm | We selected BC, DCPH, and DTNN to evaluate their suitability for long-horizon diagnosis prediction. BC with filtering strategies is commonly used but struggles to disentangle diagnosis probability from follow-up bias. DCPH, a widely used survival analysis benchmark, models log-hazard ratios but assumes a fixed relative risk over time, making it less ideal for this task. DTNN was chosen for its flexibility in capturing time-varying risk and its ability to predict diagnosis probability separately from timing. Please refer to the “Introduction” section for details. |
| (3.8) The method of evaluating model performance during training | During training, we monitored performance metrics, such as AUC_t_, to ensure the models were learning effectively. The final models with the optimal hyperparameters were selected through grid search by minimizing the validation loss (Table S5), which was expected to correspond with improved AUC_t_. These models were then evaluated on the test set. |
| (3.9) The method used for hyperparameter tuning | We used grid search for hyperparameter tuning, with the final optimal parameters, including learning rate and weight decay, detailed in Table S5. |
| (3.10) Model’s output adjustments | Please refer to Figure 2 for the model architecture and expected output format for each approach. Adjustments were made to ensure fair probability comparison between different models (see "Prediction Head" section). We also ensured that all models were calibrated, and their predicted probabilities aligned closely with observed estimates (Figure 4). |

**Evaluation**

| (4.1) Performance metrics used to evaluate the model | Model performance was primarily evaluated using AUC_t_, AP_t_ and Harrell's concordance index, which account for censoring and measure the model’s ability to discriminate between diagnosed and non-diagnosed individuals. Predicted probabilities were evaluated in the context of published trends. A more comprehensive discussion can be found in the “Performance Metrics” section. |
| --- | --- |
| (4.2) The cost or consequence of errors | Error analysis was performed by evaluating the distribution of performance using 100 bootstrap samples from the test set and computing the 95% confidence intervals for each metric. These were included in all relevant figures. |
| (4.3) The results of internal validation | We used a validation hold-out set, where grid search was applied to minimize validation loss and select the best models. The chosen models were subsequently evaluated on a separate test set. We also ensured that all models were properly calibrated, with predicted probabilities closely aligning with observed estimates (Figure 4). |
| (4.4) The final model hyperparameters | We experimented with various combinations of fully connected layers and transformer architectures, with the final model architecture shown in Figure 2. We also tuned hyperparameters, including learning rate and weight decay, with the optimal values listed in Table S5. |
| (4.5) Model evaluation on an external data set | We carefully examined the performance metrics of each model approach across different clinical conditions using held-out test sets. To reproduce the limitations of BC especially under heavy censoring, we simulated this scenario with a semi-synthetic recurrent OM dataset. In addition, we contextualized the predicted probabilities and reasoned about their correctness by comparing them with published clinical trends. For a more detailed discussion, please refer to the “Discussion” section. |
| (4.6) Characteristics relevant for detecting data shift and drift | We provided insights on out-of-distribution performance through calibration analysis (Figure 4) and year-of-birth subgroup analysis (Figure 6). These analyses highlight the impact of BC filtering strategies on out-of-distribution years, offering a closer look at data shift effects. |

**Explainability and transparency**

| (5.1) The most important features and how they relate to the outcomes | We provided empirical evidence and explanations for how different model approaches influenced outcomes across all clinical conditions. For example, we demonstrated that BC models made spurious correlations between predicted probabilities and follow-up length, leading to inflated performance in regular AUC but poorer results in AUC_t_ due to censoring. The DCPH approach, on the other hand, assumes constant relative risk and was less effective in subgroup analyses, where varying risks exist. In contrast, the DTNN approach predicted diagnosis probabilities that more accurately reflected actual clinical prevalence and temporal trends. |
| --- | --- |
| (5.2) Plausibility of model outputs | We assessed model plausibility using various metrics, including AUC, AUCt, and Harrell's concordance index, with consistent results across corresponding tables and figures. We also simulated a semi-synthetic recurrent OM dataset to replicate BC's limitations under heavy censoring. Furthermore, the DTNN approach showed predicted diagnosis probabilities that closely aligned with actual clinical prevalence and temporal trends, providing strong evidence for the plausibility of its outputs. |
| (5.3) Interpretation of a model’s results by an end user | For clinicians, TTE models provide time-based diagnosis probabilities, allowing them to assess diagnosis likelihood at various timepoints. DTNN, in particular, showed strong performance at clinically relevant operating timepoints and produced predictions that aligned with clinical prevalence and trends, aiding informed decision-making.  For ML practitioners, we present performance results to illustrate how different approaches behave across clinical conditions, highlighting BC limitations with censoring and advocating for TTE approaches like DTNN in clinical applications. |
